# Nucleolar protein NOP2/NSUN1 suppresses HIV-1 transcription and promotes viral latency by competing with Tat for TAR binding and methylation

**DOI:** 10.1371/journal.ppat.1008430

**Published:** 2020-03-16

**Authors:** Weili Kong, Ayan Biswas, Dawei Zhou, Guillaume Fiches, Koh Fujinaga, Netty Santoso, Jian Zhu

**Affiliations:** 1 Department of Pathology, Ohio State University College of Medicine, Columbus, Ohio, United States of America; 2 Gladstone Institute of Virology and Immunology, University of California, San Francisco, California, United States of America; 3 Department of Medicine, University of California San Francisco, San Francisco, California, United States of America; Loyola University Chicago, UNITED STATES

## Abstract

Recent efforts have been paid to identify previously unrecognized HIV-1 latency-promoting genes (LPGs) that can potentially be targeted for eradication of HIV-1 latent reservoirs. From our earlier orthologous RNAi screens of host factors regulating HIV-1 replication, we identified that the nucleolar protein NOP2/NSUN1, a m5C RNA methyltransferase (MTase), is an HIV-1 restriction factor. Loss- and gain-of-function analyses confirmed that NOP2 restricts HIV-1 replication. Depletion of NOP2 promotes the reactivation of latently infected HIV-1 proviruses in multiple cell lines as well as primary CD4^+^ T cells, alone or in combination with latency-reversing agents (LRAs). Mechanistically, NOP2 associates with HIV-1 5’ LTR, interacts with HIV-1 TAR RNA by competing with HIV-1 Tat protein, as well as contributes to TAR m5C methylation. RNA MTase catalytic domain (MTD) of NOP2 mediates its competition with Tat and binding with TAR. Overall, these findings verified that NOP2 suppresses HIV-1 transcription and promotes viral latency.

## Author summary

Host machinery plays a significant role in the regulation of HIV persistent infection. Identification of novel host factors kin to HIV replication and characterization of their molecular mechanisms are crucial for not only improving the fundamental understanding of host-HIV infections but also providing new drug targets for the development of novel anti-HIV therapies. The impacts of NOP2/Sun (NSUN) m5C RNA methyltransferaes (MTases) on HIV-1 infection are mainly elusive. Here, we confirmed that NOP2, NSUN m5C RNA MTase is a novel host factor suppressing HIV-1 transcription. We also identified that the potential mechanism underlying NOP2’s antiviral activities is through its competition with HIV-1 Tat protein for TAR RNA binding, which involves its RNA MTase catalytic motifs. Indeed, we also showed that NOP2 contributes to m5C methylation of TAR RNA. These findings provide new insight into the impacts of RNA MTases on HIV-1 latency.

## Introduction

Human immunodeficiency virus (HIV-1) pandemic remains a concern of global public health. Although the current combination antiretroviral therapy (cART) is potent to inhibit HIV-1 replication and reduce viral load below the detection limit, it cannot eliminate residual viremia in treated HIV+ individuals and new HIV-1infections still occur with a static rate. These HIV+ individuals have to use cART drugs for the rest of their life, while the drug resistance may develop due to the mutations of HIV-1 genomes [1]. HIV-1 undergoes latent infections mostly in resting CD4^+^ T cells despite of cART, which contributes to residual viremia and presents the major obstacle for HIV-1 elimination [2]. The host machineries play a critical role in the maintenance of HIV-1 latency since the expression of most HIV-1 genes is silenced. The 5′-long terminal repeat (LTR) of integrated HIV-1 proviruses contains the promoter/enhancer elements and is the center for determining the outcome of HIV-1 gene expression and the switch of lytic/latent stages [3], which is substantially subjected to the regulation by host machineries[4].Some of these host factors have been targeted by small-molecule compounds, which can perturb HIV-1 proviral expression and facilitate the elimination of integrated HIV-1 proviruses. Especially, the HIV-1 latency-reversing agents (LRAs), such as HDAC inhibitor (SAHA), protein kinase C agonists (bryostatin-1), and bromodomain inhibitor (JQ1) [5], have been demonstrated to reactivate latent HIV-1 and lead to its purging, which is the key of “shock and kill” HIV cure strategy. However, the efficiency of most of current LRAs is still poor, which is incapable of reversing all latently infected HIV-1 proviruses and/or inducing viral cytopathic effect sufficient for the killing of reservoir cells [6, 7]. The improved understanding of host factors regulating HIV-1 replication, especially those silencing HIV-1 viral transcription and promoting its latency, will help us to develop the more effective LRAs and other therapeutic reagents that can be applied for curing HIV.

**Fig 1:**
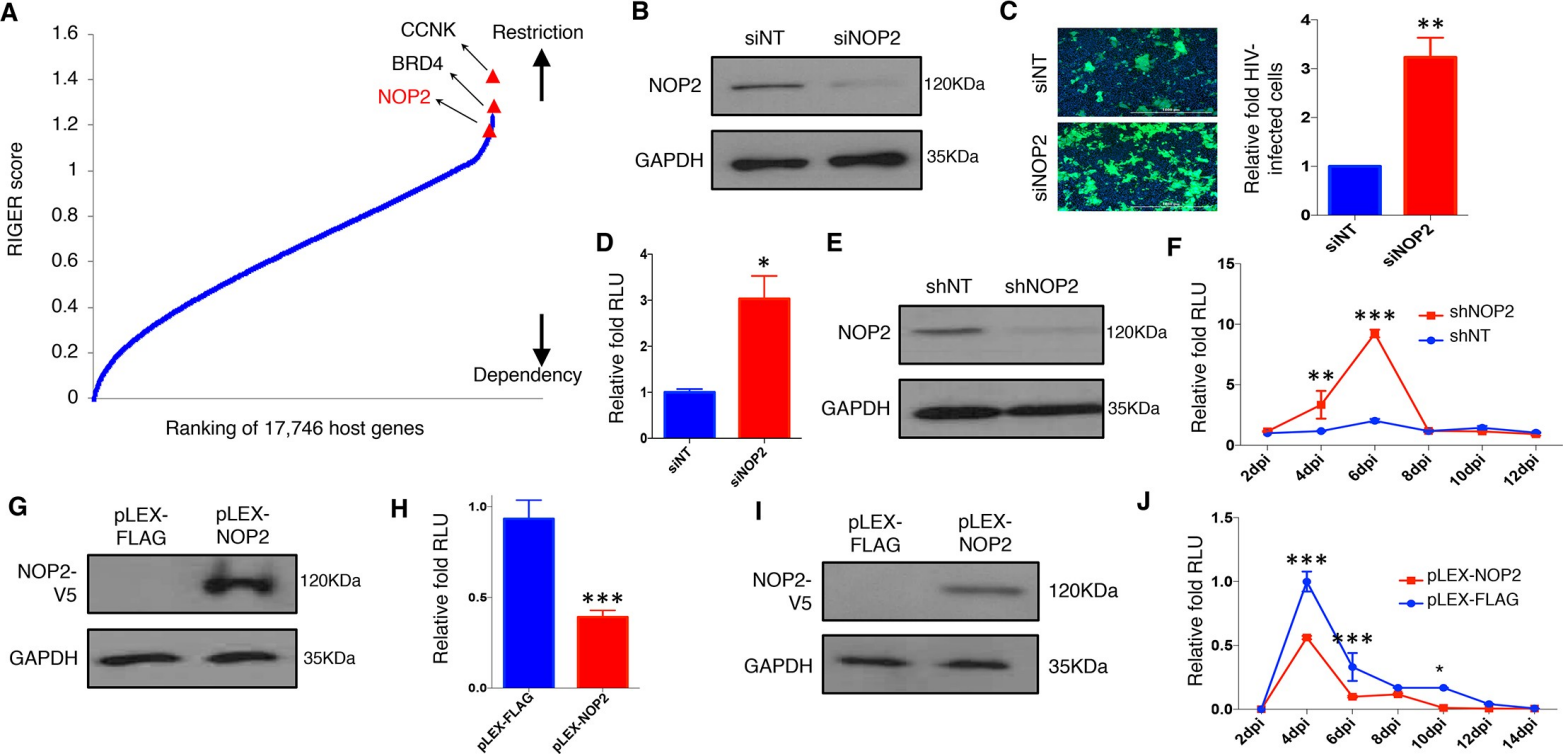
NOP2 inhibits HIV-1 replication. **(A)** RNAi gene enrichment ranking (RIGER) method was applied to analyze screens performed using multiple orthologous RNAi reagents (MORRs). Genes were ranked in order of their RIGER scores (lowest → highest), from host dependency factors to host restriction factors. RIGER analysis of these screens recognized several known host restriction factors (CCNK, BRD4) as well as new ones, such as NOP2. **(B)** MAGI-HeLa cells were transiently transfected with the indicated siRNAs (siNT or siNOP2), and NOP2 knockdown was analyzed by immunoblotting. **(C)** MAGI-HeLa cells transfected with the indicated siRNAs were infected with HIV-1 IIIB viruses, followed by the immunostaining of p24 (green). Nuclei were stained with Hoechst 33342 (blue). The infection rate is calculated by dividing p24-expressing cells by total cells, and normalized to that of non-targeting siRNA (siNT). **(D)** MAGI-HeLa cells transfected with the indicated siRNAs were infected with HIV-1 NL4–3-Luc (dEnv) viruses. The relative luminometer units (RLU) of luciferase was measured and normalized total proteins, and normalized to that of non-targeting siRNA (siNT). **(E)** Jurkat cells were stably transduced with indicated shRNAs (shNT or shNOP2) in pAPM vector, and NOP2 knockdown was analyzed by immunoblotting. **(F)** Jurkat cells stably expressing shNOP2 or shNT were infected with HIV IIIB viruses. A portion of supernatant was harvested every 2 days until 12 days post-of-infection (dpi), and titrated using the TZM-bl cells. The RLU was measured, and normalized to that of non-targeting shRNA (shNT). **(G)** MAGI-HeLa cells were stably transduced with the indicated lentiviral vectors expressing V5-tagged FLAG peptide or NOP2 ORF (pLEX-FLAG or pLEX-NOP2), and protein expression of V5-NOP2 was analyzed by immunoblotting. **(H)** MAGI-HeLa cells stably transduced with pLEX-FLAG or pLEX-NOP2 were infected with HIV-1 NL4–3-Luc (dEnv) viruses. The RLU was measured, and normalized to that of pLEX-FLAG. **(I)** Jurkat cells were stably transduced with the indicated vectors (pLEX-FLAG or pLEX-NOP2), and protein expression of V5-NOP2 was analyzed by immunoblotting. **(J)** Jurkat cells stably transduced with pLEX-FLAG or pLEX-NOP2 were infected with HIV-1 IIIB viruses. A portion of supernatant was harvested every 2 days until 14 dpi, and titrated using the TZM-bl cells. The RLU was measured, and normalized to that of pLEX-FLAG. Results were based on n = 3 experiments and presented as mean ± S.D., * p < 0.05; ** p < 0.01; *** p < 0.*001*, ANOVA.

In an earlier study, we screened 17,746 human genes using the multiple orthologous RNAi reagents coupled with integrated analytical tools for the unbiased identification of host factors regulating HIV-1 replication [8]. Along with CCNK and BRD4, two known HIV-1 restriction factors, the nucleolar protein P120 (NOP2/NSUN1) was also ranked as a top hit that restricts HIV-1 replication (Fig 1A). NOP2/NSUN1 is a nucleolar RNA-binding protein that belongs to the NOP2/SUN (NSUN) RNA-methyltransferase family, which also includes six other members: NSUN2 through NSUN7 [9]. It was recognized that NOP2 protein contains an RNA-binding domain and an RNA methyltransferase (MTase) domain [10]. The RNA-binding domain locates at the N-terminus of NOP2, which is composed of an arginine-rich region that also overlaps with a nuclear localization sequence (NLS). Interestingly, the arginine-rich region of NOP2 is similar to that of HIV Rev and Tat proteins [10]. The RNA MTase domain of NOP2 contains two catalytic motifs at the position 487-561aa [11]. Earlier studies indicate that NOP2 is a multifunctional protein, which plays an important role in cell proliferation, cell-cycle progression, tumor aggressiveness, maturation and modification of RNA, as well as chromatin organization. NOP2 is required for RNA processing and/or stability *in vivo* during preimplantation embryo development in the mouse[12]. NOP2 expresses at the higher level in the majority of human malignant tumor cells[13], and is considered as a prognostic marker for cancer aggressiveness. NOP2 also associates with the telomerase to regulate transcription of cyclin D1gene [14]. Recently, NOP2 has been found to associate with chromatins through binding with BRD4 in 5-AZA-resistant leukemia cell lines [15]. In terms of the relevance to HIV-1 study, an earlier proteomic study identified NOP2 as an RNA binding protein that associates with HIV-1 5’UTR [16]. However, the function of NOP2 regulating HIV-1 replication has never been investigated and is still not clear so far. In this study, we followed our findings from RNAi screens and confirmed the inhibitory effect of NOP2 on HIV-1 replication. We also characterized the novel function of NOP2 that silences the transcription of latently infected HIV-1 proviruses. Furthermore, we identified one potential underlying mechanism of NOP2’s silencing function, which is through the interference of HIV-1 LTR/Tat/TAR axis.

## Results

### NOP2 inhibits HIV-1 replication

To validate NOP2 as an HIV-1 host restriction factor identified from RNAi screens, we depleted the endogenous NOP2 in MAGI-HeLa cells using its siRNA (siNOP2) or non-target siRNA (siNT), whose knockdown efficiency was confirmed by immunoblotting with an anti-NOP2 antibody (Fig 1B). These cells were infected by HIV-1 IIIB viruses, and stained for HIV-1 Gag expression. Depletion of NOP2 by its siRNA increased the infection rate of HIV-1 IIIB by over 3 folds (Fig 1C). These cells were also infected with VSV-G pseudo-typed HIV-1 NL4–3-Luc (dEnv) viruses, followed by the measurement of luciferase signal (RLU). The results were similar as HIV-1 IIIB (Fig 1D), indicating NOP2’s effect is independent on the ligand-receptor mediated entry of HIV-1 virion. We further validated the effect of NOP2 in Jurkat cells depleted of endogenous NOP2 using its shRNA. NOP2 knockdown in Jurkat cells stably expressing NOP2 or non-targeting shRNA (shNOP2 or shNT) was confirmed by immunoblotting (Fig 1E). These cells were infected with HIV-IIIB viruses, and an aliquot of supernatant was collected at 2–12 days post infection (dpi) and added to TZM-bl cells for titrating newly produced HIV-1 viruses. TZM-bl indicator cell line harbors integrated luciferase and β-galactosidase genes under the control of HIV-1 5’LTR promoter, and is highly sensitive to infection of various HIV-1 strains [17]. Depletion of NOP2 by its shRNA increased HIV-1 viral production by 5–10 folds at 4, 6 dpi through the measurement of luciferase signal in TZM-bl cells (Fig 1F) or p24 expression in HIV-infected Jurkat cells by flow cytometry (S1 Fig). Such effect disappeared at 8 dpi, likely due to the obvious cytotoxicity induced by increased viral titer (S1 Fig).

Alternatively, we also determined the impact of NOP2 overexpression on HIV-1 replication. We transiently transfected pQCXIP-NOP2 (HA-tagged) or empty vector into HEK293 cells, followed by the infection of VSV-G pseudo-typed HIV-1 NL4–3-Luc (dEnv) viruses. Overexpression of NOP2 in HEK293 cells reduced luciferase signal by ~40% (S2 Fig). We also stably transduced MAGI-HeLa and Jurkat cells with pLEX-NOP2 (V5-tagged) or the control vector (pLEX-FLAG expressing FLAG peptide). NOP2 overexpression was confirmed by immunoblotting with an anti-V5 antibody (Fig 1G and 1I). Overexpression of NOP2 in MAGI-HeLa cells reduced the infection of VSV-G pseudo-typed HIV-1 NL4-3-Luc (dEnv) viruses by ~50% (Fig 1H). Similarly, overexpression of V5-NOP2 (pLEX vector) in Jurkat cells reduced the production of HIV-1 IIIB viruses by ~50% at 4, 6, 10 dpi compared to the empty vector, measured through the TZM-bl titration (Fig 1J). Taking together, NOP2 was confirmed as an HIV-1 restriction factor.

### NOP2 suppresses HIV-1 transcription

**Fig 2:**
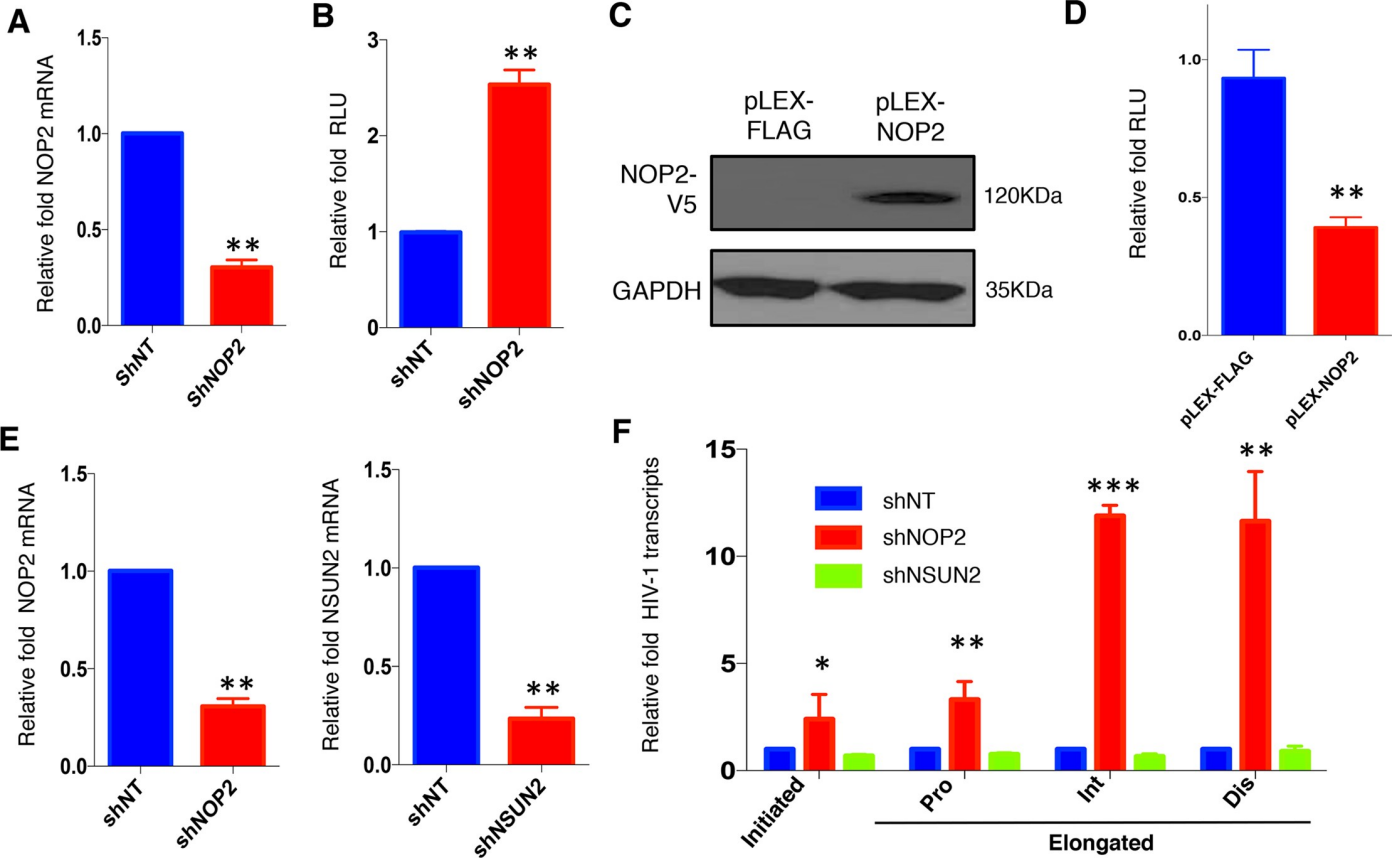
NOP2 suppresses HIV-1 transcription. **(A)** TZM-bl cells stably expressing FLAG-tagged HIV-1 Tat protein in the retroviral vector pQXCIP (pQCXIP-Tat) were transiently transduced with the indicated shRNA (shNT or shNOP2) in pAPM vector, and NOP2 knockdown was analyzed by reverse transcription coupled qPCR (RT-qPCR). **(B)** The RLU of cells in (A) was measured, and normalized to that of shNT. **(C)** HEK293 cells were stably transduced with the indicated vectors (pLEX-FLAG or pLEX-NOP2), and protein expression of V5-NOP2 was analyzed by immunoblotting. **(D)** The cells in **(C)** were transiently co-transfected with pcDNA-Tat, HIV-LTR-Luciferase, and pTK-Renilla vectors, followed by the dual-luciferase reporter assay. The RLU (luciferase/renilla) was measured, and normalized to that of pLEX-FLAG. **(E)** Jurkat cells were stably transduced with the indicated shRNA (shNT, shNOP2, or shNSUN2) in pAPM vector, and NOP2 or NSUN2 knockdown was analyzed by RT-qPCR. **(F)** The cells in **(E)** were infected with HIV-1 IIIB viruses, followed by the RNA extraction and RT-qPCR to measure HIV-1 initiated or elongated (proximal [Pro], intermediate [Int], and distal [Dis]) transcripts. The level of each HIV-1 transcript was normalized to that of shNT. Results were based on n = 3 experiments and presented as mean ± S.D., * p < 0.05; ** p < 0.01; *** p < 0.*001*, ANOVA.

We next determined which step of HIV-1 life cycle is repressed by NOP2. We transiently transduced pAPM vector expressing shNOP2 or shNT into the TZM-bl cells stably expressing FLAG-tagged HIV-1 Tat protein (TZM-bl-Tat). Depletion of NOP2 in TZM-bl-Tat cells was confirmed by RT-qPCR (Fig 2A), which increased the Tat/LTR-driven luciferase signal by 2–3 fold (Fig 2B). We also prepared the HEK293 cells stably transduced with pLEX-NOP2 or pLEX-FLAG, and NOP2 overexpression was confirmed by immunoblotting (Fig 2C). These cells were transiently co-transfected with pcDNA-Tat, HIV-LTR-luciferase, and TK-renilla vectors. Luciferase signal was measured and normalized by renilla signal measured from the same cell samples as previously described [18]. Overexpression of NOP2 reduced the normalized LTR signal by over 60% (Fig 2D), further supporting that the presence of NOP2 suppresses Tat/LTR-driven gene expression.

Recently, there was a report showing that the NSUN2, another member from the same RNA MTase family of NOP2, is involved in HIV-1 viral protein translation through m5C methylation of HIV-1 RNA genome [19]. We thus prepared Jurkat cells stably expressing shRNAs of NOP2, NSUN2 (shNOP2, shNSUN2), or shNT, whose knockdown efficiency was confirmed by RT-qPCR (Fig 2E). We determined the effect of NOP2 or NSUN2 depletion on the level of HIV-1 initiated and elongated transcription in these cells infected with HIV-1 IIIB viruses by RT-qPCR using primer sets targeting initial, proximal, intermediate, and distal sites of HIV-1 transcripts as previously described [20]. Depletion of NOP2, but not NSUN2, moderately increased the initial and proximal HIV-1 transcripts by less than 5 folds while potently increased the intermediate and distal HIV-1 transcripts by over 10 folds (Fig 2F). Overall, these results indicated that NOP2 suppresses HIV-1 transcription, preferentially the transcriptional elongation.

### NOP2 facilitates HIV-1 latency

**Fig 3:**
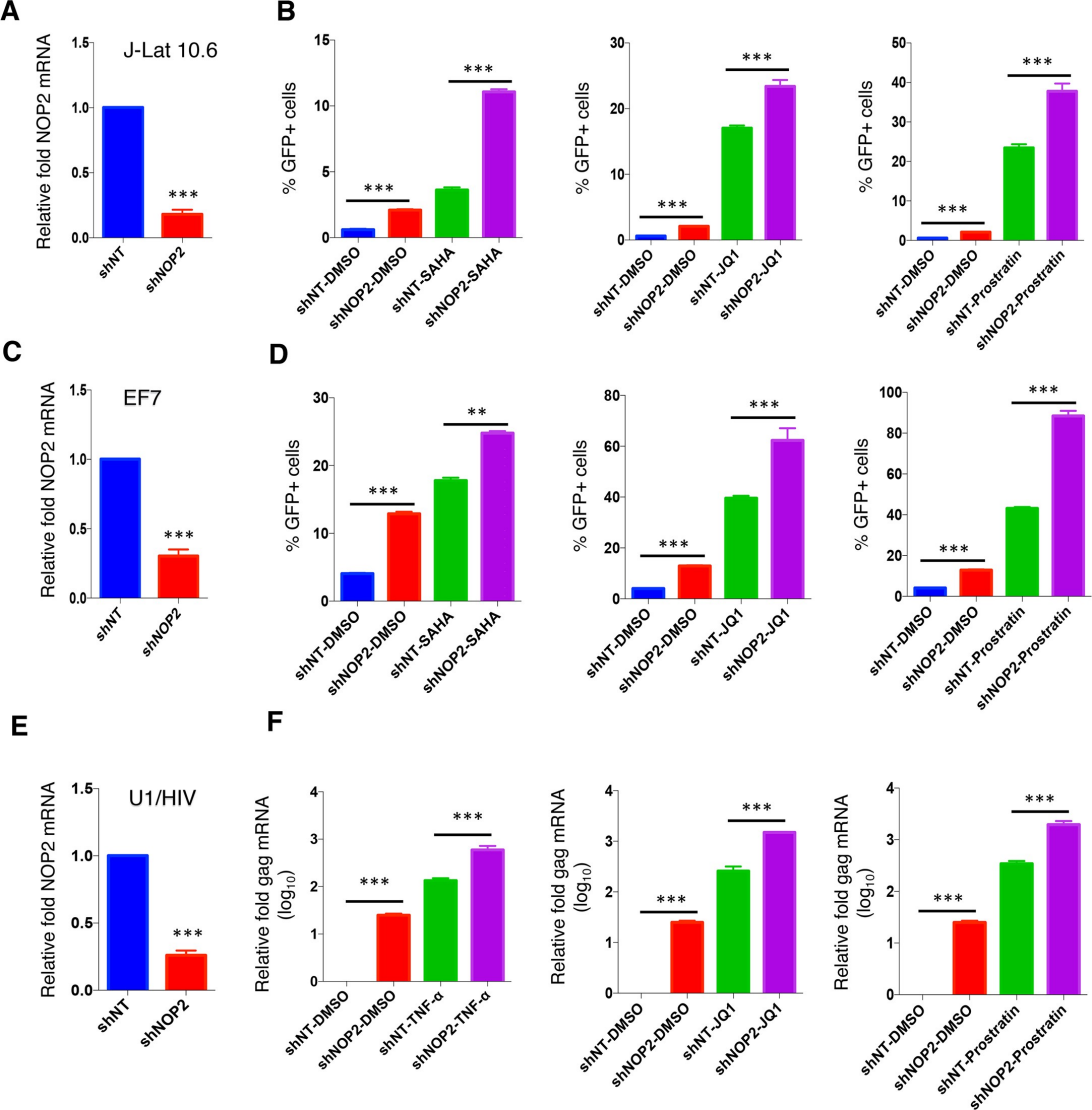
Knockdown of NOP2 benefits HIV-1 reactivation in multiple latency cell lines. **(A, C, E)** Jurkat-based HIV-1 latency cell lines, J-Lat 10.6 **(A)** and EF7 **(C)**, as well as a monocytic one, U1/HIV **(E)**, were stably transduced with the indicated shRNA (shNT or shNOP2) in pAPM vector, and NOP2 knockdown was analyzed by RT-qPCR. **(B, D)** Jurkat-based J-Lat 10.6 **(B)** and EF7 **(D)** cells stably expressing shNT or shNOP2 were treated with DMSO, SAHA (1 uM), JQ1 (0.5 uM), or Prostratin (0.5 uM), to reactivate latent HIV-1. Percentage of GFP-expressing cells was determined by flow cytometry. **(E)** U1/HIV cells stably expressing shNT or shNOP2 were stimulated with DMSO, TNF-α (10 ng/ml), JQ1 (0.5 uM), or Prostratin (0.5 uM), to reactivate latent HIV-1. Total RNA was extracted, followed by RT-qPCR to measure the mRNA level of HIV-1 Gag, which was normalized to that of shNT. Results were based on n = 3 experiments and presented as mean ± S.D., * p < 0.05; ** p < 0.01; *** p < 0.*001*, ANOVA.

Since NOP2 suppresses HIV-1 transcription, we next determined whether the presence of NOP2 is required for the maintenance of HIV-1 latency. Initially, we determined the effect of NOP2 depletion or overexpression on HIV-1 latency using J-Lat A2 cells that harbor the integrated “LTR-Tat-IRES-GFP” minigenome. J-Lat A2 cells stably expressing shNOP2 or shNT, or stably expressing pLEX-NOP2 or pLEX-FLAG, were treated with DMSO, or LRAs (JQ1, SAHA), followed by the flow cytometry analysis of GFP expression indicating HIV-1 reactivation. Depletion of NOP2 indeed increased the rate of HIV-1 reactivation by 2–3 folds at either basal (DMSO) or stimulated with JQ1 or SAHA, while overexpression of NOP2 moderately blocked HIV-1 reactivation at these conditions (S3 Fig). Depletion of NOP2 only weakly facilitated Prostratin-induced HIV-1 reactivation in J-Lat A2 cells (S3 Fig). We also further confirmed the impact of NOP2 depletion on HIV-1 reactivation in several other latency cell lines containing nearly full length of HIV-1 proviruses, including J-Lat 10.6, EF7, and U1/HIV cells (Fig 3). J-Lat 10.6 cells are Jurkat cells latently infected with a full length HIV-1 genome (HIV-R7) with a non-functional Env due to a frameshift and GFP in place of the Nef gene. EF7 T cells are Jurkat cells latently infected with the HIV-1 NL4-3 genome with EGFP placed between the Env and Nef genes, which is integrated into the WHSC1 gene in the converse-sense orientation relative to the transcriptional direction of the host gene [21–23]. U1/HIV is a subclone of pro-monocyte U937 cells latently infected with two copies of integrated HIV genomes containing Tat mutations with a point mutation, H13L, of one Tat and missing of the ATG initiation codon of the other Tat [24]. NOP2 knockdown efficiency in these cells stably expressing shNOP2 or shNT was confirmed by RT-qPCR (Fig 3A, 3C and 3E). Depletion of NOP2 not only significantly increased the rate of HIV-1 reactivation in J-Lat 10.6 and EF7 cells (by measuring GFP-positive cell percentage, S4 Fig) and U1/HIV cells (by measuring relative HIV gag mRNA) at the basal level (DMSO), but also enhanced the latency-reversing potency of LRAs (SAHA, JQ1, or Prostratin) in all these cells (Fig 3B, 3D and 3F). Depletion of NOP2 resulted in a similar effect in another two latency cell lines, J89GFP and THP89GFP, that are Jurkat T cells and THP1 monocytic cells latently infected with an alternative HIV-1 molecular clone, 89.6, expressing EGFP [22] (S5 Fig).

**Fig 4:**
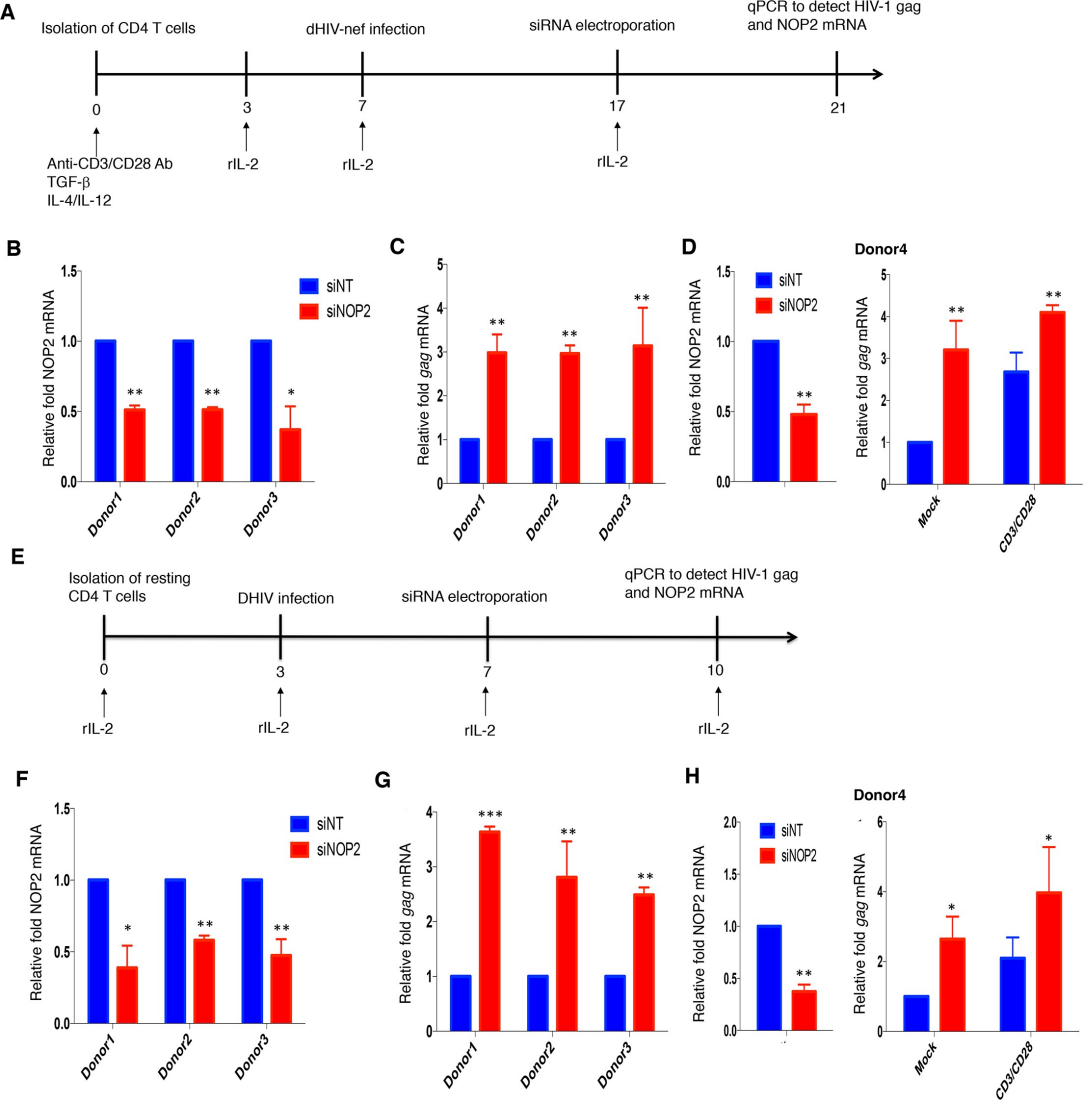
Validation of NOP2’s effect on HIV-1 latency using primary CD4^+^ T cell models. **(A)** A primary cell model of HIV-1 latency based on the use of activated CD4^+^ T cells. **(B, C)** Primary CD4^+^ T cells isolated from three donors (donors 1–3) were activated and infected with VSV-G pseudo-typed dHIV-nef. Once cells returned back to quiescent stage and HIV-1 latency was established, these cells were transiently transfected with the indicated siRNA (siNT or siNOP2) through electroporation, followed by the total RNA extraction and RT-qPCR to measure the knockdown of NOP2 **(B)** and expression of HIV-1 Gag **(C)**, which was normalized to that of siNT. **(D)** Primary CD4^+^ T cells isolated from the additional donor (donor 4) was subjected to the same treatment as in **(B, C)**, except that HIV-1 reactivation was analyzed by measuring Gag mRNA level at both the basal level with mock treatment and the stimulation condition using anti-CD3/28 antibodies on Dynabeads. **(E)** A primary cell model of HIV-1 latency based on the use of resting CD4^+^ T cells. **(F, G)** Resting CD4^+^ T cells isolated from three donors (donors 1–3) were spinoculated with VSV-G pseudotyped dHIV-nef. Cells were transiently transfected with the indicated siRNA (siNT or siNOP2) through electroporation. Total RNAs were extracted from these cells and analyzed by RT-qPCR to measure the mRNA level for NOP2 **(F)** or HIV-1 Gag **(G)**, which was normalized to that of siNT. **(H)** Primary CD4^+^ T cells isolated from the additional donor (donor 4) was subjected to the same treatment as in **(F, G)**, except that HIV-1 reactivation was analyzed by measuring Gag mRNA level at both the basal level with mock treatment and the stimulation condition using anti-CD3/28 antibodies on Dynabeads. Results were based on n = 3 replicates and presented as mean ± S.E.M., * p < 0.05; ** p < 0.01; *** p < 0.*001*, ANOVA.

Although we confirmed the role of NOP2 in the maintenance of HIV-1 latency using multiple cell lines, it would be better to use the primary CD4^+^ T cells for establishment of HIV-1 latency given that they are more physiologically relevant. We employed two primary CD4^+^ T cell models of HIV-1 latency for evaluation of NOP2. First, we evaluated NOP2 using the primary CD4^+^ T cell model of HIV-1 latency previously described by Vicente Planelles’ group [25]. Naïve CD4^+^ T cells were isolated and treated with anti-CD3/anti-CD28 antibodies, as well as TGF-β, anti-IL-4, and anti-IL-12 antibodies, to generate non-polarized, memory CD4^+^ T cells. These cells were spinoculated with VSV-G pseudo-typed, Env-deleted, dHIV-nef viruses. HIV-1 latency was established during a long-term culture (Fig 4A). siRNAs targeting NOP2 (siNOP2) or the non-targeting control (siNT) were delivered into these cells through electroporation. siRNA-mediated NOP2 knockdown efficiency and the reactivated HIV-1 Gag expression were measured by RT-qPCR. Across primary CD4^+^ T cells from three HIV-negative donors, siNOP2 led to the moderate NOP2 knockdown (Fig 4B), which effectively induced HIV-1 gene expression (Fig 4C). Second, we utilized the primary resting CD4^+^ T cell model of HIV-1 latency established by Una O’Doherty’s group [26] (Fig 4E). The resting CD4^+^ T cells were isolated from naïve primary CD4^+^T cells, and directly infected with dHIV-nef viruses through spinoculation. Similarly, siRNAs (siNOP2 or siNT) were delivered into these cells through electroporation, followed by the measurement of NOP2 and HIV-1 Gag mRNA level. Consistently, siNOP2-mediated NOP2 knockdown promoted HIV-1 gene expression in this model, using primary resting CD4^+^ T cells from three HIV-negative donors (Fig 4F and 4G). Furthermore, we showed that depletion of NOP2 by its siRNA increases the HIV-1 Gag mRNA level not only at the basal level but also at the condition of anti-CD3/CD28 stimulation in the above two HIV-1 latency models using the CD4^+^ T cells isolated from the fourth donor (Fig 4D and 4H). In summary, our data clearly showed that NOP2 is required for maintenance of HIV-1 latency and that its depletion benefits HIV-1 reactivation at both basal and LRA-stimulated conditions.

### NOP2 binds with HIV-1 TAR RNA at 5’LTR and leads to its m5C methylation

**Fig 5:**
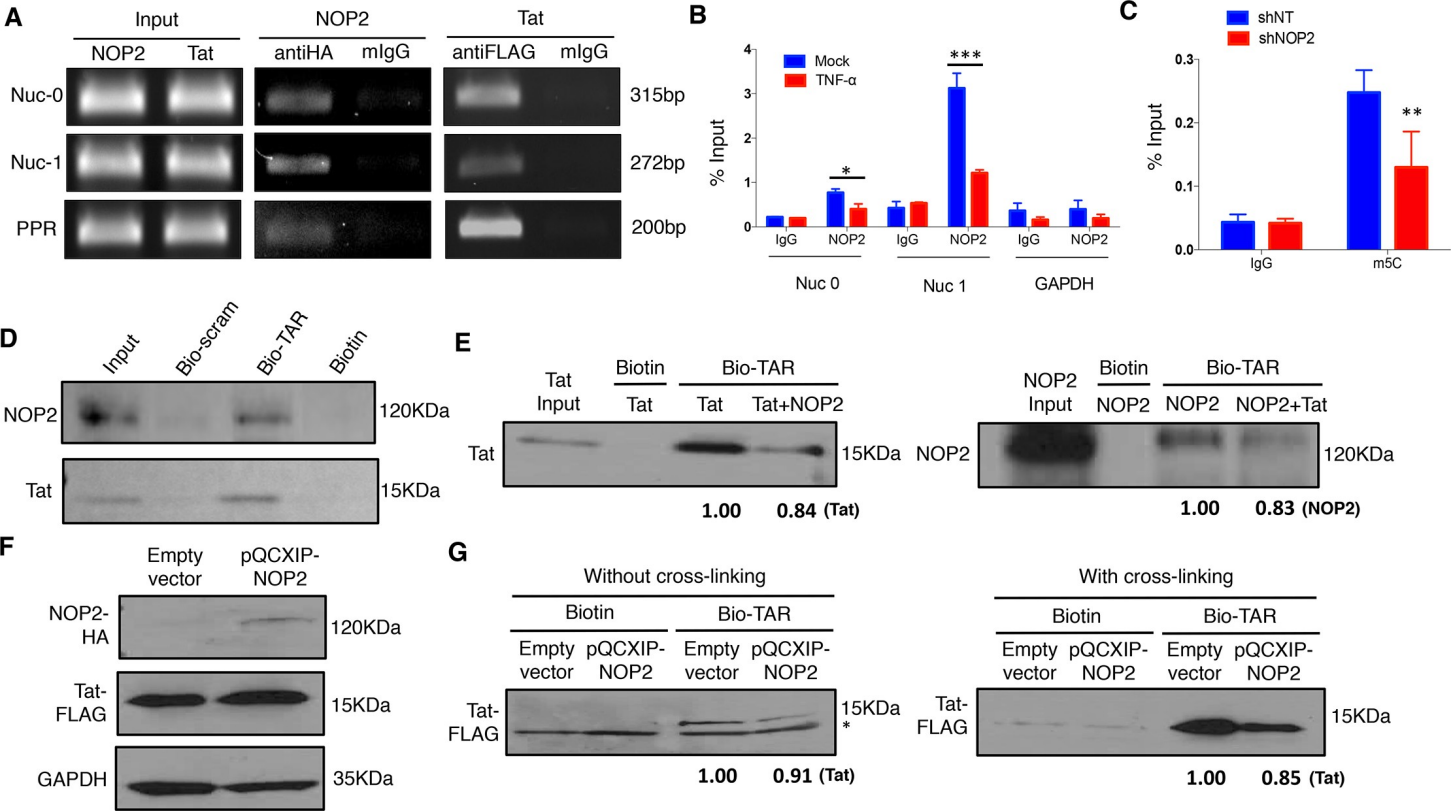
NOP2 binds with HIV-1 TAR RNA and contributes to its m5C methylation. **(A)** TZM-bl cells were transiently transfected with either pQCXIP-NOP2 (HA-tagged) or PQCXP-Tat (FLAG-tagged). Cells were cross-linked using formaldehyde. Cell lysates were prepared and split to halves for incubation with mouse anti-HA/FLAG antibody or mouse IgG (mIgG). Co-precipitated DNA samples were analyzed by semi-quantitative PCR using primer sets that amplify HIV-1 5’ LTR region (Nuc0, Nuc1, PPR). **(B)** J-Lat A2 cells were treated with or without TNF-α (10 ng/ml). Lysates from cross-linked cells were prepared and split to halves for incubation with mouse anti-NOP2 antibody or mIgG. Co-precipitated DNA samples were analyzed by qPCR using primer sets that amplify Nuc0, Nuc1, or the negative control GAPDH DNA sequence. The DNA level of Nuc0, Nuc1 and GAPDH amplicon was determined as the percentage of input (1% of lysate). **(C)** HEK293 cells stably expressing the indicated shRNA (shNT or shNOP2) were transiently transfected with the pU16TAR plasmid for expression of HIV-1 TAR RNA. Cell lysates were prepared and treated with DNase I, followed by total RNA extraction. Extracted RNAs were incubated with an anti-m5C antibody or control IgG. Immuno-precipitated RNA samples were analyzed by RT-qPCR using the primer set that amplifies HIV-1 TAR. The DNA amplicon of TAR was determined as the percentage of input (1% of lysate). Results in **(B)** and **(C)** were based on n = 3 experiments and presented as mean ± S.D., * p < 0.05; ** p < 0.01; *** p < 0.*001*, ANOVA. **(D)** Recombinant NOP2 or Tat protein (6xHis-tagged) was purified from bacteria, and incubated with the equal amount of synthesized biotinylated TAR RNA (bio-TAR), its scrambled RNA (bio-scram), or the free biotin *in vitro*. The protein-RNA complex was affinity-precipitated using streptavidin magnetic beads. The input or precipitated Tat or NOP2 protein was analyzed by immunoblotting. **(E)** Recombinant Tat protein was incubated with bio-TAR in the presence (1:1 molar ratio) or absence of recombinant NOP2 protein, or with free biotin *in vitro*. Vise versa, recombinant NOP2 protein was incubated with bio-TAR in the presence (1:1 molar ratio) or absence of recombinant Tat protein, or with free biotin *in vitro*. In both cases, the protein-RNA complex was affinity-precipitated using streptavidin magnetic beads. The input or precipitated Tat or NOP2 protein was analyzed by immunoblotting, and the relative intensity of pulled down Tat or NOP2 was calculated. **(F)** TZM-bl cells stably expressing FLAG-tagged HIV-1 Tat protein in the retroviral vector pQXCIP (pQCXIP-Tat) were transiently transduced with pQCXIP-NOP2 (HA-tagged) or empty vector. Protein expression of HA-NOP2 and FLAG-Tat was analyzed by immunoblotting. **(G)** Cells in **(F)** were either without (left panel) or with (right panel) cross-linking. Cell lysates were prepared and incubated with bio-TAR or free biotin *in vitro*. The protein-RNA complex was affinity-precipitated using streptavidin magnetic beads. The input or precipitated Tat protein was analyzed by immunoblotting, and the relative intensity of pulled down Tat was calculated. * A non-specific protein band beneath FLAG-Tat at the “without cross-linking” condition was noted.

An earlier study indicated that NOP2 contains the arginine-rich RNA-binding domain similar as HIV-1 Rev and Tat proteins [10]. Furthermore, another study identified that NOP2 is an HIV-1 5’ UTR associated RNA binding protein (RBP) through the RNA affinity chromatography [16]. Therefore, we postulated that the HIV-1 transcriptional silencing activity of NOP2 is through the interference of HIV-1 LTR/Tat/TAR axis. First, we confirmed that NOP2 associates with the HIV-1 5’ LTR region (Nuc0, Nuc1, PPR) by ChIP-PCR using an anti-V5 mouse antibody or mouse IgG (mIgG) as a negative control in TZM-bl cells transiently transfected with pQCXIP vector expressing HA-NOP2 (Fig 5A). In parallel, we performed ChIP-PCR for Tat in TZM-bl cells transiently transfected with pQCXIP-Tat (FLAG-tagged) as a positive control. ChIP-PCR for endogenous NOP2 using an anti-NOP2 or IgG antibodies (ChIP-grade) in J-Lat A2 cells treated with or without TNFα further confirmed the association of NOP2 with 5’LTR region (Nuc0, Nuc1) but not the negative control GAPDH DNA sequence, and also revealed that TNFα treatment leads to NOP2 dissociation from this region, likely favoring HIV-1 reactivation (Fig 5B). Second, we determined whether NOP2 contributes to the m5C RNA methylation of HIV-1 TAR RNA due to that NOP2 belongs to the NOP2/SUN RNA methyl-transferase family [27]. We transiently transfected the pU16 vector expressing HIV-1 TAR RNA in HEK293 cells that were stably transduced with shNOP2 or shNT. Total RNAs were extracted from these cells and subjected to the immuno-precipitation (IP) of 5C-methylated RNAs using an anti-m5C antibody or IgG (the negative control) as previously described [28]. The IPed RNAs were converted to cDNA and subjected to RT-qPCR using the primer set that amplifies HIV-1 TAR sequence. Indeed, depletion of NOP2 resulted in the reduction of TAR m5C RNA methylation by ~50% (Fig 5C). We expect that NOP2-mediated m5C RNA methylation of HIV-1 TAR may affect its normal function required for HIV-1 transcription, supported by the earlier studies showing that m5C methylation of non-coding RNAs affects their binding with proteins as well as their turnovers [29, 30].

Lastly, we experimentally determined the physical interaction of NOP2 with HIV-1 TAR RNA based on the prediction that NOP2 may do so due to its arginine-rich RNA-binding domain similar as Tat [10]. For this, we incubated the recombinant NOP2 or Tat protein (6xHis-tagged) with the synthesized biotinylated TAR (bio-TAR), biotinylated scrambled RNA (bio-scram), or the free biotin *in vitro*, followed by the pull-down assays using the streptavidin magnetic beads. The immunoblotting assays for the precipitated protein samples showed that both NOP2 and Tat proteins readily interact with bio-TAR but not bio-scram RNA nor free biotin (Fig 5D). We further performed the *in vitro* TAR pull-down assays for Tat with or without the presence of NOP2 or *vise versa*. The presence of NOP2 reduced Tat-TAR interaction, while the presence of Tat reciprocally reduced NOP2-TAR interaction as well (Fig 5E), indicating that NOP2 and Tat compete with each other for TAR binding. We also performed the competition binding assays using the cell lysates. TZM-bl cells stably expressing FLAG-tagged Tat protein were transiently transfected with the pQCXIP-NOP2 (HA-tagged) and the empty vector. Expression of HA-NOP2 and FLAG-Tat proteins in TZM-bl cells were confirmed by immunoblotting (Fig 5F). Cell lysates were prepared and incubated with bio-TAR or free biotin either without or with cross-linking, followed by the pull-down assays using the streptavidin magnetic beads. Overexpression of NOP2 indeed reduced the interaction of FLAG-Tat protein with bio-TAR, especially at the cross-linking condition (Fig 5G). These results undoubtedly illustrated that NOP2 binds with HIV-1 TAR RNA, interfering with Tat-TAR interaction that is critical for HIV-1 transcription. This provides the explanation of NOP2’s silencing effect on HIV-1 proviral expression.

### NOP2 MTD domain competes with Tat for binding with TAR

**Fig 6:**
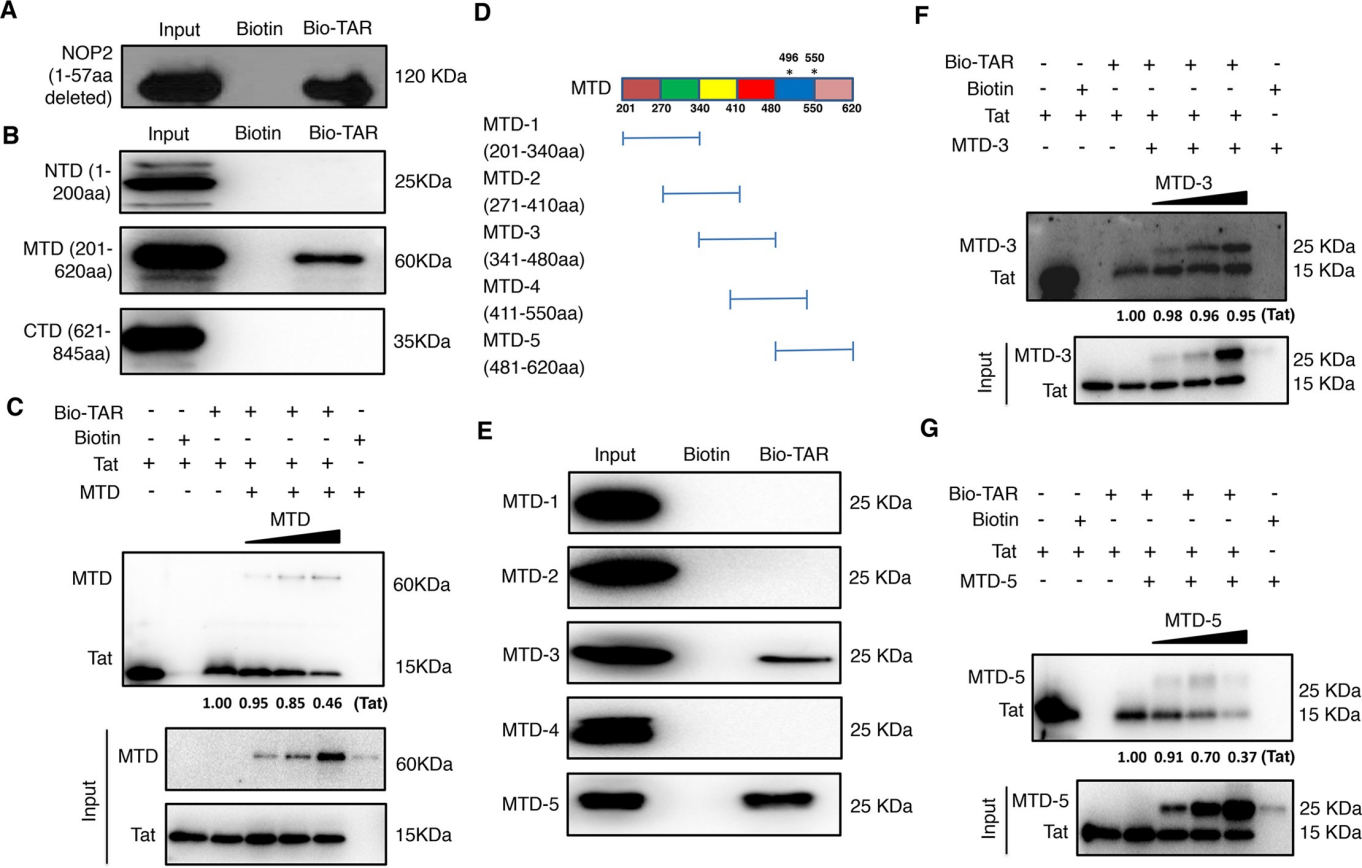
The MTD domain of NOP2 competes with Tat for TAR binding. **(A)** Recombinant NOP2 protein with deletion of 1–57 amino acids (6xHis-tagged) was purified from bacteria, and incubated with bio-TAR or free biotin *in vitro*. The protein-RNA complex was affinity-precipitated using streptavidin magnetic beads. The input or precipitated NOP2 (1-57aa deleted) protein was analyzed by immunoblotting. **(B)** Recombinant NOP2 protein domains, NTD (1-200aa), MTD (201-620aa), and CTD (621-845aa), were purified from bacteria, and incubated with bio-TAR or free biotin *in vitro*, followed by the affinity precipitation using streptavidin magnetic beads. The input or precipitated NOP2 protein domains (NTD, MTD, CTD) were analyzed by immunoblotting. **(C)** Recombinant Tat protein was incubated with bio-TAR in the absence or presence of NOP2 MTD domain at the increased doses, or with free biotin *in vitro*, followed by the affinity precipitation using streptavidin magnetic beads. The input or precipitated Tat or NOP2 MTD domain was analyzed by immunoblotting. The relative intensity of pulled down Tat was calculated. **(D)** NOP2 MTD domain was further divided into five smaller domains (MTD-1 to 5), which were ~140aa with 70aa overlap. The positions of two catalytic cysteine residues (496aa, 550aa) in the MTD were indicated by asterisks (*). **(E)** Recombinant NOP2 MTD smaller domains, MTD-1 to 5, were purified from bacteria, and incubated with bio-TAR or free biotin *in vitro*, followed by the affinity precipitation using streptavidin magnetic beads. The input or precipitated smaller MTD domains of NOP2 were analyzed by immunoblotting. **(F, G)** Recombinant Tat protein was incubated with bio-TAR in the absence or presence of NOP2 MTD-3 **(F)** or MTD-5 **(G)** domain at the increased amount, or with free biotin *in vitro*, followed by the affinity precipitation using streptavidin magnetic beads. The input or precipitated Tat or smaller MTD domain of NOP2 was analyzed by immunoblotting. The relative intensity of pulled down Tat was calculated.

Following the finding that NOP2 binds with TAR, we further mapped which domain(s) of NOP2 mediates such interaction. Initially, we deleted the first 57 amino acids (1-57aa) at the N-terminus of NOP2, which is the predicted RNA-binding region similar as Tat [10]. The recombinant NOP2 protein with deletion of 1-57aa still binds with bio-TAR but not free biotin (Fig 6A). This is an intriguing finding, indicating that NOP2 binds with TAR through other domains/regions. We then divided NOP2 protein into three domains, NTD (N-terminal domain, 1-200aa), MTD (RNA MTase catalytic domain, 201-620aa), and CTD (C-terminal domain, 621-845aa), as previously described [31]. From the *in vitro* TAR-binding assay, we identified that only NOP2 MTD domain binds with bio-TAR but not free biotin (Fig 6B). This result seems consistent with the finding that NOP2 contributes to m5C methylation of TAR RNA (Fig 5C). The *in vitro* Tat-TAR binding was also determined with the presence of recombinant NOP2 MTD domain at the increased doses. The immunoblotting assays showed that NOP2 MTD domain competes with Tat for TAR binding in a dose-dependent manner (Fig 6C). MTD is still a relatively large domain, so we further divided it into five smaller domains (MTD-1 to 5), each of which is ~140aa with 70aa overlap (Fig 6D). From the *in vitro* TAR-binding assay, we identified that both MTD-3 and MTD-5 domains of NOP2 bind with bio-TAR but not free biotin (Fig 6E). Interestingly, MTD-5 possesses two complete RNA MTase catalytic motifs (Fig 6D)[31]. The *in vitro* Tat-TAR competition binding assays further unrevealed that although both MTD-3 and MTD-5 of NOP2 bind with TAR in a dose-dependent manner only MTD-5 competes with Tat for TAR binding (Fig 6F and 6G). Overall, these results shed the light in the molecular details of NOP2-TAR interaction that is mediated by two binding sites (MTD-3 and MTD-5) of NOP2, and that MTD-5 containing the RNA MTase catalytic motifs competes with Tat for TAR binding. Binding of MTD-3 with TAR is likely through other site(s).

## Discussion

**Fig 7:**
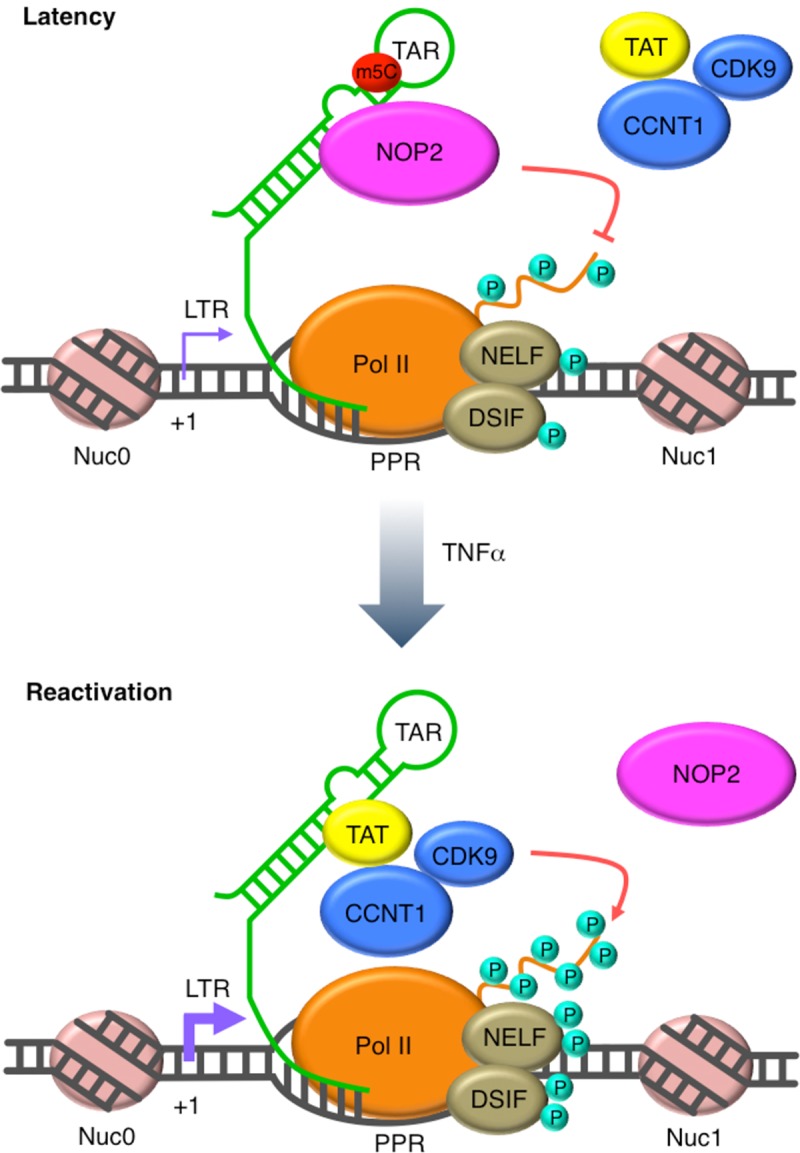
A tentative model illustrates NOP2’s silencing effect on HIV-1 proviral expression. At the latent phase of HIV-1 infection, NOP2 occupies at the 5′ LTR of HIV-1 proviruses, binds with HIV-1 TAR RNA through its methylation domain and leads to its m5C methylation. HIV-1 Tat protein is a critical viral factor that recruits the host positive transcription elongation complex b (P-TEFb), composed of cyclin T1 (CCNT1) and CDK9, to activate RNA polymerase II (Pol II) for HIV-1 transcriptional elongation. The binding of NOP2 with TAR competes with Tat-TAR interaction, preventing the recruitment of Tat and P-TEFb as well as the activation of Pol II. Overall, the association of NOP2 at 5’ LTR suppresses HIV-1 transcriptional elongation. Once latent HIV-1 proviruses are reactivated, for example, by TNFα, NOP2 dissociates from the 5′ LTR of HIV-1 proviruses. Thus, Tat binds with TAR and recruits P-TEFb for activation of Pol II, promoting HIV-1 transcriptional elongation.

Our comprehensive RNAi screens allowed us to unbiasedly identify host factors regulating HIV-1 replication. Since we scored and ranked host genes based on weighing the parallel screening results of multiple orthologous siRNA reagents for the same gene, we proved that this strategy significantly improves the success rate of following-up validation experiments[8]. We focused on NOP2 due to the emerging role of RNA methylation in regulating HIV-1 infections epitranscriptionally [19, 32]. Recently, NSUN2 was shown to mediate m5C methylation of HIV-1 RNA genomes, which led to the increase of HIV-1 viral protein translation, while this study indicated that NOP2 has no such effect [19]. Therefore, it is surprising that NOP2 was identified as a potential host restriction factor of HIV-1 from our RNAi screens, assuming that m5C RNA methylation overall favors HIV-1 replication. However, our own results showed that depletion of NSUN2 by RNAi has no impact on HIV-1 transcription (Fig 2E and 2F) but NOP2 plays a suppressive role (Fig 2A–2D), indicating that NSUN2 and NOP2 perhaps affect different aspects of HIV-1 life cycle. Indeed, Courtney *et al* investigated the m5C methylation of HIV-1 RNA genomes mainly mediated by NSUN2 but not NOP2 [19], while we found that NOP2 contributes to the m5C methylation of HIV-1 short TAR RNA hairpin (Fig 5C). Therefore, our findings are not necessarily contradictory to those from Courtney *et al* [19], which rather unravel the complicated roles of m5C RNA methylation in regulating HIV-1 replication that is executed by multiple m5C RNA MTases. A reconciled model would be that NSUN2 mediates m5C methylation of HIV-1 RNA genomes and benefits viral protein synthesis, while NOP2 mediates m5C methylation of HIV-1 TAR and interferes with proviral transcription (Fig 7).

Our following-up studies revealed that NOP2 blocks HIV-1 replication (Fig 1) through suppression of LTR/Tat-driven HIV-1 transcription (Fig 2). Consistent with these findings, we further confirmed that the presence of NOP2 promotes HIV-1 latency and prevents its reactivation in multiple CD4^+^ T cell or monocyte based latency cell lines (Fig 3) as well as two primary CD4^+^ T cell models (Fig 4). Interestingly, depletion of NOP2 not only reverses HIV-1 latency by its own, but also synergizes with the use of multiple LRAs to promote HIV-1 reactivation. This suggests that HIV-1 reactivation induced by NOP2 depletion may use a mechanism independent of those host machineries either triggered or inhibited by these LRAs. Indeed, our in-depth mechanistic analysis unraveled a previously unreported, unique activity of NOP2 that competes with HIV-1 Tat for TAR binding (Figs 5 and 6) and is quite different as the mechanisms of tested LRAs. This is consistent with the finding that NOP2 preferentially blocks HIV-1 transcriptional elongation (Fig 2E and 2F), since it is known that Tat-TAR interaction is critical for recruitment of the positive transcription elongation factor, P-TEFb, which activates RNA polymerase II (Pol II) for its engagement in HIV-1 transcriptional elongation[33]. A recent study also showed that the protein chimera between the CycT1-binding domain of Tat and the RNA-binding domain and the Cdk9-inhibitory domain of HEXIM1 inhibits HIV-1 transcription by competing with Tat for TAR binding [28], further supporting that Tat-TAR binding is critical. In addition, our finding that NOP2 associates with HIV-1 5’ LTR region (Fig 5A) supports the NOP2-TAR interaction as the potential mechanism for NOP2 to silence HIV-1 proviruses. It is also consistent with the earlier finding that NOP2 associates with HIV-1 5’ UTR where TAR locates as an RNA-binding protein [16]. Furthermore, we recognized that NOP2 dissociates from Nuc-0 and Nuc-1 chromatin regions of 5’ LTR upon HIV-1 reactivation induced by TNF-α, indicating that NOP2’s suppressive effect needs to be removed to favor the resurrected HIV-1 gene expression. Indeed, it has been shown by another study that NOP2 associates with the chromatin structure to regulate gene expression through BRD4 and RNA Pol II [15, 34]. Meanwhile, we showed that the endogenous protein level of NOP2 in HIV-1 latency cell line J-Lat 10.6 is similar as that in the parental Jurkat cells (S6 Fig).

NOP2 is one of the “writers” that add methyl group to mostly tRNAs [27]. Our results showed that NOP2 participates in the m5C methylation of TAR, a small HIV-1 RNA hairpin (Fig 5C). Our in-depth analysis also confirmed that NOP2 binding with HIV-1 TAR RNA is not through the predicted arginine-rich RNA-binding region of NOP2 [10], but rather through its two MTD regions (MTD-3 and MTD-5) (Fig 6D and 6E). However, only MTD-5 contains two complete RNA MTase catalytic motifs around two cysteine residues at 496aa and 550aa, and competes with Tat for TAR binding (Fig 6F and 6G). Although MTD-4 also contains two catalytic cysteine residues (496aa, 550aa), the flanking residues at the second cysteine are cut short (Fig 6D), explaining why MTD-4 failed to bind with TAR (Fig 6E). The supportive evidence is that the NTD of yeast NOP2 containing the RNA-binding domain binds with 25S rRNA but not yeast tRNAs or HIV-1 derived RNAs [31]. Based on these findings, the tentative working model is that NOP2 binds with TAR through its RNA MTase domains, which competes with Tat and leads to m5C methylation of TAR (Fig 7) resulting in the consequence of HIV-1 transcriptional suppression. Although we can conclude that the competition between NOP2 and Tat is most likely due to the common binding site on TAR, it is still not clear what is the impact of NOP2-mediated m5C methylation of TAR on HIV-1 proviral expression. Although the functional significance of m5C RNA methylation remains elusive so far, it occurs to various RNA types, including tRNAs, rRNAs, mRNAs, as well as long and smaller non-coding RNAs (ncRNAs), which fine-tune the RNA processing, stability, translation, as well as RNA-protein interaction [27,32]. To determine the exact role of NOP2-meidated TAR methylation, it requires the more substantial effort to pinpoint which site(s) of TAR is methylated by NOP2. Furthermore, the “readers” of m5C RNA methylation could be involved as well, which modulates the functions and activities of TAR RNA. MBD2, a protein binder of m5C DNA or RNA that belongs to the methyl-CpG binding domain family, was shown to regulate HIV-1 latency by recruiting the NuRD to HIV-1 LTR promoter [35]. It is possible that certain RNA methylation “reader(s)”, pending for identification, may contribute to the maintenance of HIV-1 latency through the binding with HIV-1 TAR RNA at 5’ LTR promoter.

## Materials and methods

### Viruses

VSV-G pseudo-typed HIV-1 NL4-3-luciferase (NL4-3-Luc) virus (Cat. 3418) from the NIH AIDS reagent program and the DHIV (dEnv) virus kindly provided by Vicente Planelles (University of Utah) were generated as previously described [36]. Lentiviruses expressing NOP2 or non-targeting (NT) shRNAs in pAPM vector (pAPM-shNOP2, pAPM-shNT), retroviruses expressing HIV-1 Tat (FLAG-tagged) and NOP2 (HA-tagged) in pQCXIP vector (pQCXIP-Tat, pQCXIP-NOP2) were generated as previously described[37]. HIV-1 IIIB wild-type virus was kindly provided by the NIH AIDS reagent program and propagated accordingly [38].

### Cells

The following cell lines were received from the NIH AIDS reagent program: Jurkat Clone E6-1 (Cat. #177), J-Lat A2 (Cat. #9854) and 10.6 (Cat. #9849), U1/HIV (Cat. #165). HIV-1 latently infected T cell line EF7 was kindly provided by Olaf Kutsch (University of Alabama) [39]. J89GFP and THP89GFP HIV-1 latency cell lines were kindly provided by David Levy (New York University). All T cell lines were maintained in RPMI-1640 medium supplemented with 10% FBS. CD4^+^ T cells isolated from healthy donors were purchased from LONZA (Cat. #70025), and cultured in complete media (RPMI-1640, 10% FBS, 1 × glutamine, 1 × MEM non-essential amino acid solution, 20 mM HEPES) containing 30 IU/ml IL-2. MAGI-HeLa, TZM-bl, and HEK293T fembryonic kidney cells were cultured in Dulbecco's modified Eagle's medium (DMEM) supplemented with 10% fetal bovine serum (FBS), penicillin (100 U/ml), and streptomycin (100 μg/ml).

### Antibodies

ChIP grade mouse anti-NOP2 (Cat. # sc-398884X) and IgG (Cat. #sc2025) antibodies were obtained from Santa Cruz Biotechnology. Anti-FLAG (Cat. #2368) antibody was purchased from Cell Signaling Technology. Mouse Anti-V5 (Cat. # R960-25), anti-HA (Cat. # 26183) and anti-NOP2 (Cat. #PA5-34712) antibodies were purchased from Thermo Fisher Scientific. Antibodies against CD3 (Cat. # 16-0037-85) and CD28 (Cat. # 16-0289-85) were purchased from eBioscience. IL-12 (Cat. # AB-219-NA) and IL-4 (Cat. # AB-204-NA) antibodies were purchased from R&D System. An anti-m5C antibody (Cat. # C15200081) was purchased from Diagenode. HIV-1 Gag p24 IgG1 monoclonal antibody was produced from hybridoma cell line (NIH AIDS reagent program).

### Plasmids

A NOP2 cDNA from pDONR223 was cloned into pET-DEST42 vector through Gateway LR recombination reaction (Thermo Fisher Scientific). Truncated NOP2 domains (1-57aa deletion; NTD, 1-200aa; MTD, 201-620aa; CTD, 621-845aa; MTD-1, 201-340aa; MTD-2, 271-410aa; MTD-3, 341-480aa; MTD-4, 411-550aa; MTD-5, 481-620aa) and HIV-1 Tat were also cloned into pET-DEST42. A NOP2 cDNA with an N-terminal HA tag was cloned into the retroviral vector pQCXIP using Agel and PacI sites. shNOP2 and shNT hairpins were synthesized by IDT and cloned into the lentiviral vector pAPM using XhoI and EcoRI sites. pcDNA-Tat, HIV-LTR-luciferase, and pTK-Renilla vectors used for dual luciferase assays were described previously [18].

### Proteins

pET-pDEST42 vector containing NOP2 ORFs, full-length or truncated domains, or Tat ORF was transformed into Rosetta™(DE3) *E*. *coli* strain for protein expression. Bacteria cells were incubated in the LB media at 37°C until the optical density (OD) reached ~0.6 at 600nm. Protein expression was induced by treating bacteria cells with 0.1mM IPTG for 16 h at 16°C. Bacteria cells were then harvested and re-suspended in 1x lysis buffer [50 mM NaH_2_PO_4_ (PH 8.0), 500 Mm NaCl] on ice. Lysozyme (8 mg) was added into the mixture, which was incubated on ice for 30 mins, followed by brief sonication. Cell debris were removed by centrifugation at 12,000x g for 10 min at 4°C. The supernatant was loaded onto 3-mL HisTrap column of the His-tagged fusion protein purification system (Thermo Fisher Scientific, Cat. #88226), which was washed with the provided binding buffer for 3 times. His-tagged proteins were eluted using the provided elution buffer. Protein concentration was measured using the protein assay kit.

### RNA synthesis

Biotinylated TAR and its scrambled RNA was synthesized by IDT. The TAR sequence is 5’/5Biosg/rGrGrGrUrCrUrCrUrCrUrGrGrUrUrArGrArCrCrArGrArUrCrUr GrArGrCrCrUrGrGrGrArGrCrUrCrUrCrUrGrGrCrUrArArCrUrArGrGrGrArArCrCrC-3’ according to the earlier reports[40]. The scrambled RNA sequence is 5’/5Biosg/rArCrCrGrUrCrGrCrUrGrGrUrCrCrGrGrCrUrArUr CrGrCrUrArArCrArUrGrGrArCrArCrGrUrGrGrUrArGrCrArUrUrCrGrArArGrCrUrGrCrUrGrU-3’.

### Compounds

The following HIV-1 latency-reversing agents (LRAs) were used: JQ1 (SML-1524; Sigma-Aldrich), Prostratin (SC-203422; Santa Cruz Biotechnology), SAHA (SC-220139; Santa Cruz Biotechnology), bryostatin-1 (SC-201407; Santa Cruz Biotechnology). TGF-β1 (7754-BH-005), IL-12 (419-ML-010) and IL-4 (204-IL-050) were purchased from R&D System. TNF-α (T0157) and IL-2 (SRP3085) were purchased from Sigma-Aldrich.

### siRNAs and shRNAs

For siRNA assays, NOP2 siRNA (s9611) or non-targeting control (NT) siRNA (AM4636) were from Thermo Fisher Scientific. MAGI cells were seeded in the 12-well plate, and transiently transfected with siNOP2 or siNT using RNAiMAX reagents or through electroporation by using the Neon™ Transfection System (Thermo Fisher Scientific) according to the manufacturer's instruction. For shRNA assays, lentiviral vector pAPM expressing shNOP2 or shNT were transduced into the Jurkat, J-Lat 10.6, EF7, J89GFP, THP89GFP, U1/HIV cells followed by the puromycin selection. shNOP2 sequence:5’-AAAAGACTGGACTAGTGGTGTA-3’; shNUSN2 sequence: 5’- ACCCCTGCATCATGGTGGTCAA -3’; shNT sequence: 5’- CACAAACGCTCTCATCGACAAG -3’.

### Chromatin immunoprecipitation (ChIP) assay

ChIP assay was conducted as described previously [37, 41]. Cells were cross-linked by using 0.5% formaldehyde, followed by treating with 125 mM glycine to quench the reaction. After washing with cold 1× PBS, cells were lysed for 10 min on ice in 1× CE buffer (10 mM HEPES-KOH [pH 7.9], 60 mM KCl, 1 mM EDTA, 0.5% Nonidet P-40, 1 mM DTT and protease inhibitor tablet). The nuclei were pelleted by centrifugation at 700 × *g* for 10 min at 4°C and re-suspended in 1× SDS lysis buffer (1% SDS, 10 mM EDTA, 50 nM Tris-HCl [pH 8.1] and protease inhibitor mixture). Nuclear lysates were sonicated for 2 min to fragment genomic DNAs, and subsequently diluted to 10-fold with 1× ChIP buffer (0.01% SDS, 1% Triton X-100, 1.2 mM EDTA, 16.7 mM Tris-HCl [pH 8.1], 150 nM NaCl and protease inhibitor mixture). The lysates were incubated overnight at 4°C with specific antibodies or control mouse IgG or rabbit IgG. Protein A/G beads (Invitrogen) were pre-blocked with 0.5 mg/ml BSA and 0.125 mg/ml calf thymus DNA for 1 h at 4°C, and then added to the lysate-antibody mixture for another incubation at 4°C for 2 h. IPed samples were washed with the following buffers: low salt buffer (0.1% SDS, 1% Triton X-100, 2 mM EDTA, 20 mM Tris-HCl, pH 8.1, 150 mM NaCl); high-salt buffer (0.1% SDS, 1% Triton X-100, 2 mM EDTA, 20 mM Tris-HCl, pH 8.1, 500 mM NaCl); LiCl buffer (0.25 M LiCl, 1% NP-40, 1% Na-deoxycholate, 1 mM EDTA, 10 mM Tris-HCl [pH 8.1]); and 1× TE buffer (10 mM Tris-HCl [pH 8.1], 0.1 mM EDTA)], and were eluted with 1× elution buffer (1% SDS, 0.1 M NaHCO_3_) at room temperature. To reverse the cross-linking, the eluted samples were incubated at 65°C overnight in the presence of 0.2 M NaCl. The eluted samples were then treated with proteinase K, and the DNA species were precipitated by using phenol–chloroform. The DNA pellets were re-suspended in water and quantified by semi-PCR and qPCR. Input (1%) was used for qPCR analysis. PCR primers: Nuc-0 (F:5′- GAA GGG CTA ATT TGG TCC *CA* -3′; R: 5′- GAT GCA GCT CTC GGG CCA TG -3′), L1(PPR) (F: 5′- CGA GAG CTG CAT CCG GAG TA -3′; R: 5′- AGC TTT ATT GAG GCT TAA GC -3′), Nuc-1 (F:5′- AGT GTG TGC CCG TCT GT -3′; R: 5′- TTG GCG TAC TCA CCA GTC GC -3′), GAPDH (F:5'-GGG TGT GAA CCA TGA GAA GT-3'; R:5'-GTA GAG GCA GGG ATG TT-3').

### Primary CD4^+^ T cell models of HIV-1 latency

We utilized the Planelles’ model with slight modifications [25]. In brief, CD4^+^ T cells were activated using CD3/CD28 antibodies in the presence of TGF-β1 (10 ng/ml), anti-human IL-12 (2 μg/ml), and anti-human IL-4 (1 μg/ml) for 3 days. Cells were then spinoculated with VSV-G pseudo-typed HIV-1 DHIV (dEnv) viruses at 1,200 × g for 2 hr at 25°C. Cells were kept in culture for another 10 days to allow HIV-1 to establish latency, followed by the transient transfection of siNOP2 or siNT into cells by electroporation. We also employed the O'Doherty’s model with slight modification [26]. Briefly, resting CD4^+^ T cells were isolated from naïve primary CD4^+^ T cells. Cells were cultured in presence of IL-2 (30U/mL) for 3 days, and spinoculated with VSV-G pseudo-typed HIV-1 DHIV (dEnv) viruses at 1,200 × g for 2 h at 25°C. Cells were transiently transfected with siNOP2 or siNT by electroporation. In both models, cells were harvested at 3 days post of siRNA transfection with the mock treatment (donors 1–4) or with anti-CD3/CD28 stimulation (donor 4), and subjected to RNA extractions and RT-qPCR assays to measure the NOP2 knockdown and HIV-1 *gag* gene expression.

### Real-time qPCR

Total RNAs were extracted from the assayed cells by using the RNeasy kit (Qiagen), and 0.2–1 μg of RNA was reversely transcribed using the iScript™ cDNA Synthesis Kit (Bio-Rad). Real-time PCR assay was conducted using the SYBR Premix ExTaq II (Bio-Rad) and gene-specific primers. The PCR reactions were performed on a Bio-Rad CFX connect qPCR machine under the following conditions: 95°C for 10 min, 40 cycles of 95°C for 15 s and 60°C for 1 min. Relative percentage of gene expression was normalized to the GAPDH control, and was calculated using the formula: 2 ^(Δ C T of gene−Δ CT of GAPDH)^. RT-qPCR primers: NOP2(F:5'- GAGTTGCTCCTGAGTGCTATT-3',R:5'-GAAACAGCCACACCTACAA ATG-3'), HIV-1 Gag (F: 5'- GAC GCT CTC GCA CCC ATC TC -3'; R: 5'- CTG AAG CGC GCA CGG CAA -3'), NSUN2 (F: 5'- AGG AAC TGG TGA CAC AGA AAT AG-3'; R:5'- TCT AAG GAG TTG CGG AAT GTG-3'), GAPDH (F:5'- GCC TCT TGT CTC TTA GAT TTG GTC -3'; R: 5'- TAG CAC TCA CCA TGT AGT TGA GGT -3').

### Immunostaining assay

MAGI-HeLa or Jurkat cells were infected with HIV-1 virus, then fixed in 4% paraformaldehyde in PBS (Thermo Fisher Scientific) for 10 min at room temperature and permeabilized with 0.1% Triton X-100 in PBS for 15 min. Following permeabilization, cells were treated with block buffer 5% FBS in PBS for 1 h at room temperature. Cells were incubated with primary antibodies diluted in block buffer overnight at 4°C. Cells were washed 3 times with PBS, each for 10 min, followed by incubation with Alexa Fluor-conjugated secondary antibody (Life Technologies) in block buffer for 1 h at room temperature. These cells were either imaged on Cytation 5 cell imaging multi-mode reader (BioTek) and analyzed using Gen5 software (MAGI-HeLa) [42], or subjected to the flow cytometry analysis (Jurkat).

### Immunoblotting assays

Cell pellets were homogenized in 1X RIPA containing protease inhibitor cocktail, and incubated on ice for 30 min. The cell lysate was cleared by centrifugation at 13,000rpm for 30 min. The protein concentration was measured by BCA kit (Thermo Fisher Scientific), then boiled in the 2× SDS loading buffer, and analyzed by immunoblotting. Protein samples were separated by SDS-PAGE, and transferred to PVDF. Blots were blocked with 5% skimmed milk in PBS and probed with anti-V5, anti-Flag or anti-NOP2 primary antibodies followed by anti-mouse HRP-conjugated secondary antibodies. Protein bands were visualized with ECL Plus chemiluminescence reagent.

### Streptavidin pull-down

The biotinylated TAR RNA (bio-TAR) or its scrambled RNA (bio-scram) was dissolved in the RNA folding buffer (100 mmol/L KCl, 20 mmol/L HEPES [pH 7.6], and 5 mmol/L MgCl_2_), and incubated at 70°C for 2 min and cooled at room temperature. 50 pmol of HIV-1 Tat or NOP2 (full-length or specific domain) protein was incubated with 50 pmol of bio-TAR, bio-scram, or free biotin with 40 U of RNasin OUT RNase inhibitors at room temperature for 30 min. Dynabeads M-280 Streptavidin beads (Thermo Fisher Scientific) were washed twice with each of following solutions: solution A (0.1 mol/L NaOH and 0.05 mol/L NaCl), solution B (0.1 mol/L NaCl), and RNA binding buffer (100 mmol/L KCl, 20 mmol/L HEPES [pH 7.6], 5 mmol/L MgCl2, 10% glycerol, 1 mmol/L DTT, 0.1% IGEPAL^®^, and 400 μmol/L RVC), and then re-suspended in 400 μL RNA binding buffer. Washed beads were incubated with the protein-RNA mixtures at room temperature for 2 h. The beads were washed 5 times with RNA binding buffer through magnetic separation. Protein samples were treated with 2× SDS buffer at 95°C for 10 min and ready for immunoblotting assays.

### Flow cytometry

J-Lat A2, J-Lat 10.6, EF7, THP89GFP, J89GFP cells, with or without NOP2 depletion by its shRNA, were cultured in a 48-well plate at 1 × 10^5^ cells per well in a total volume of 200 μl RPMI-1640 media supplied with 10% FBS. Cells were treated with JQ1 (1 μM), SAHA (0.5 μM), Prostratin (1 μM), TNF-α (10ng/ml), or DMSO for 24 h. GFP-positive cells were gated and analyzed by flow cytometry using an Accuri C6 plus Flow Cytometer (BD).

## Supporting information

S1 Fig Jurkat cells stably expressing shNOP2 or shNT were infected with HIV-1 IIIB viruses. At the indicated days post infection (dpi), cells were harvested and divided into two portions. One portion was subjected to the immunostaining of HIV-1 Gag p24 by using the anti-p24 mouse antibody, followed by the flow cytometry analysis (A). The other portion was subjected to the cell viability assay using the LIVE/DEAD™ Fixable Far Red Dead Cell Stain Kit (Invitrogen, CA) following the manufacturer’s instruction (B).
(PDF)

S2 Fig (A) TZM-bl cells stably expressing FLAG-tagged HIV-1 Tat protein in the retroviral vector pQXCIP (pQCXIP-Tat) were transiently transduced with pQCXIP-NOP2 (HA-tagged) or empty vector. (B) For cells in (A), the RLU of luciferase was measured, and normalized to that of empty vector.
(PDF)

S3 Fig (A) J-Lat A2 cells stably expressing the indicated shRNA (shNT or shNOP2) were stimulated with DMSO, JQ1 (0.5 uM), SAHA (1 uM), or Prostratin (0.5 uM), to reactivate latent HIV-1. Percentage of GFP-expressing cells was determined by flow cytometry, and normalized to that of shNT. (B) J-Lat A2 cells stably transduced with pLEX-FLAG or pLEX-NOP2 were stimulated with DMSO, JQ1 (0.5 uM), or SAHA (1 uM), to reactivate latent HIV-1. Percentage of GFP-expressing cells was determined by flow cytometry, and normalized to that of pLEX-FLAG. * p < 0.05; ** p < 0.01; *** p < 0.*001*, ANOVA.
(PDF)

S4 Fig (A, B) The HIV-1 latency cell lines, J-Lat 10.6 (A) or EF7 (B), were stably transduced with the indicated shRNA (shNT or shNOP2) in pAPM vector. These cells were stimulated with DMSO, JQ1 (0.5 uM), SAHA (1 uM), or Prostratin (0.5 uM), to reactivate latent HIV-1. Percentage of GFP-expressing cells was determined by flow cytometry.
(PDF)

S5 Fig (A, B) The additional HIV-1 latency cell lines, J89GFP (A) or TH89GFP (B), were stably transduced with the indicated shRNA (shNT or shNOP2) in pAPM vector. These cells were stimulated with DMSO, JQ1 (0.5 uM), SAHA (1 uM), or Prostratin (0.5 uM), to reactivate latent HIV-1. Percentage of GFP-expressing cells was determined by flow cytometry.
(PDF)

S6 Fig (A) Endogenous protein level of NOP2 in HIV-1 latency cell line J-Lat 10.6 and the parental Jurkat cells was measured by immunoblotting.
(PDF)
