# Supplementary figures and images for: Nucleolar protein NOP2/NSUN1 suppresses HIV-1 transcription and promotes viral latency by competing with Tat for TAR binding and methylation
Source: PLoS Pathog. 2020 Mar 16;16(3):e1008430. doi: 10.1371/journal.ppat.1008430 (PMC7098636; doi:10.1371/journal.ppat.1008430)

Figure S1

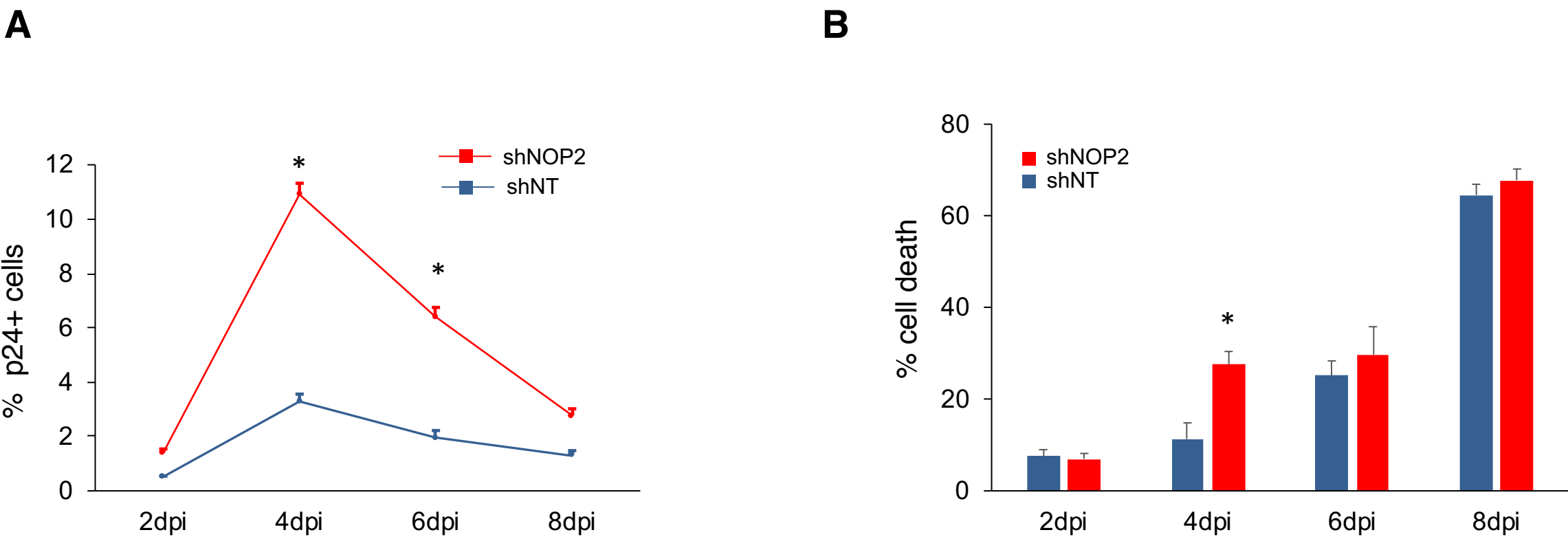

Supplement: S1 Fig — At the indicated days post infection (dpi), cells were harvested and divided into two portions. One portion was subjected to the immunostaining of HIV-1 Gag p24 by using the anti-p24 mouse antibody, followed by the flow cytometry analysis (A). The other portion was subjected to the cell viability assay using the LIVE/DEAD™ Fixable Far Red Dead Cell Stain Kit (Invitrogen, CA) following the manufacturer’s instruction (B). (PDF) [file ppat.1008430.s001.pdf]

Figure S2

A

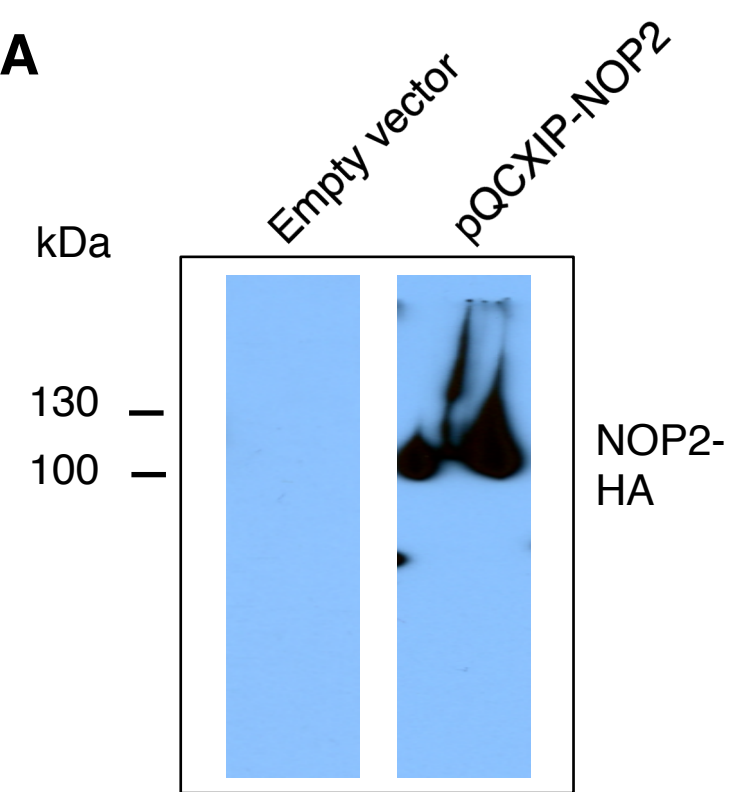

B

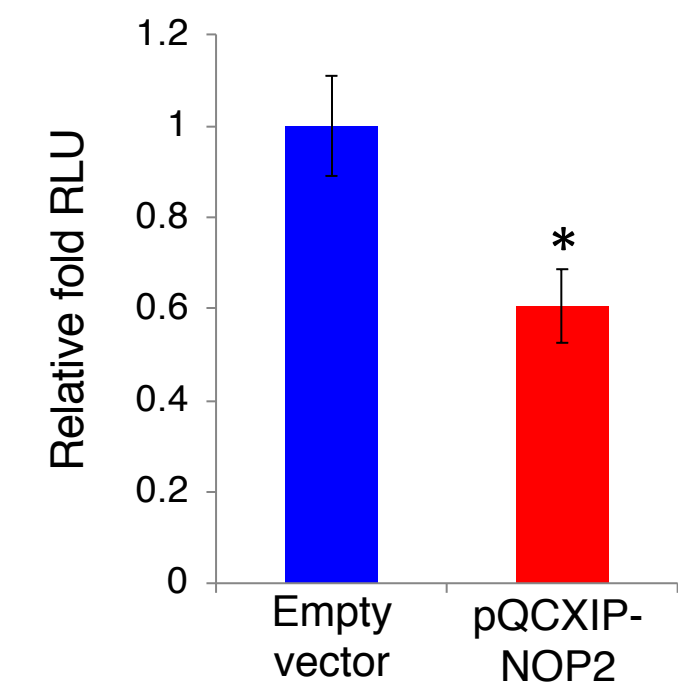

Supplement: S2 Fig — (A) TZM-bl cells stably expressing FLAG-tagged HIV-1 Tat protein in the retroviral vector pQXCIP (pQCXIP-Tat) were transiently transduced with pQCXIP-NOP2 (HA-tagged) or empty vector. (B) For cells in (A), the RLU of luciferase was measured, and normalized to that of empty vector. (PDF) [file ppat.1008430.s002.pdf]

Figure S3

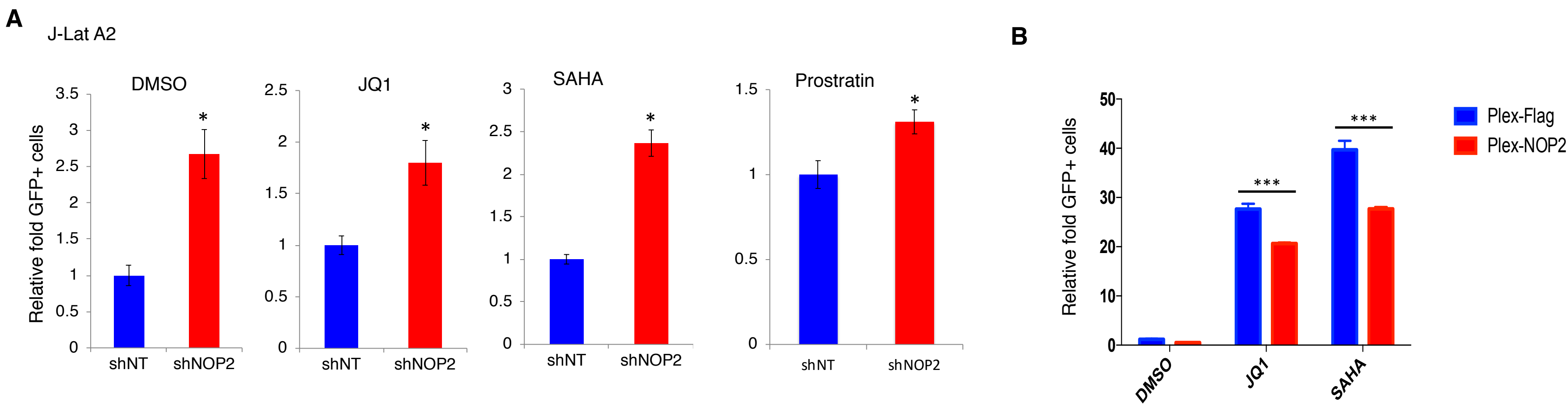

Supplement: S3 Fig — (A) J-Lat A2 cells stably expressing the indicated shRNA (shNT or shNOP2) were stimulated with DMSO, JQ1 (0.5 uM), SAHA (1 uM), or Prostratin (0.5 uM), to reactivate latent HIV-1. Percentage of GFP-expressing cells was determined by flow cytometry, and normalized to that of shNT. (B) J-Lat A2 cells stably transduced with pLEX-FLAG or pLEX-NOP2 were stimulated with DMSO, JQ1 (0.5 uM), or SAHA (1 uM), to reactivate latent HIV-1. Percentage of GFP-expressing cells was determined by flow cytometry, and normalized to that of pLEX-FLAG. * p < 0.05; ** p < 0.01; *** p < 0.001, ANOVA. (PDF) [file ppat.1008430.s003.pdf]

Figure S4

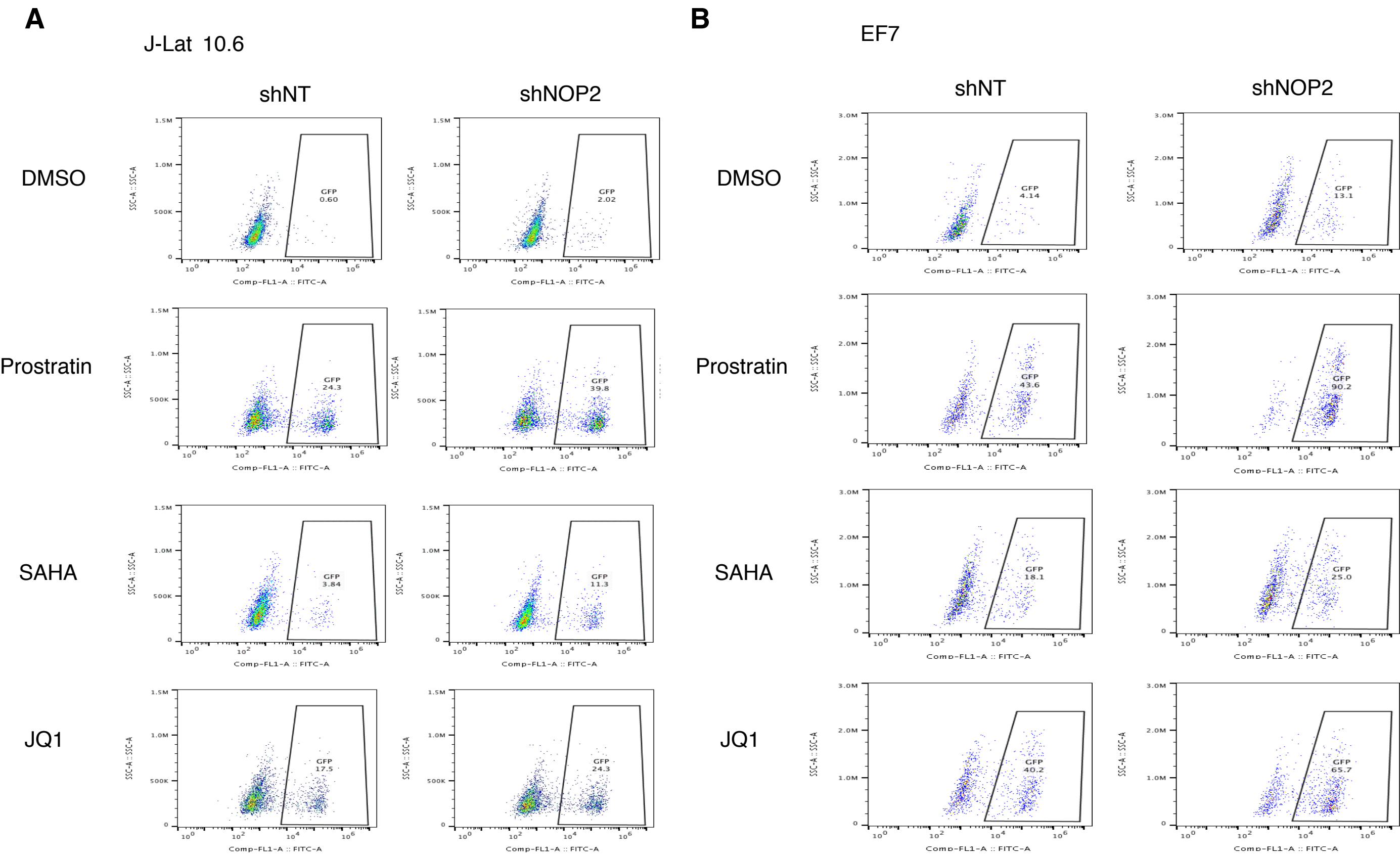

Supplement: S4 Fig — (A, B) The HIV-1 latency cell lines, J-Lat 10.6 (A) or EF7 (B), were stably transduced with the indicated shRNA (shNT or shNOP2) in pAPM vector. These cells were stimulated with DMSO, JQ1 (0.5 uM), SAHA (1 uM), or Prostratin (0.5 uM), to reactivate latent HIV-1. Percentage of GFP-expressing cells was determined by flow cytometry. (PDF) [file ppat.1008430.s004.pdf]

Figure S5

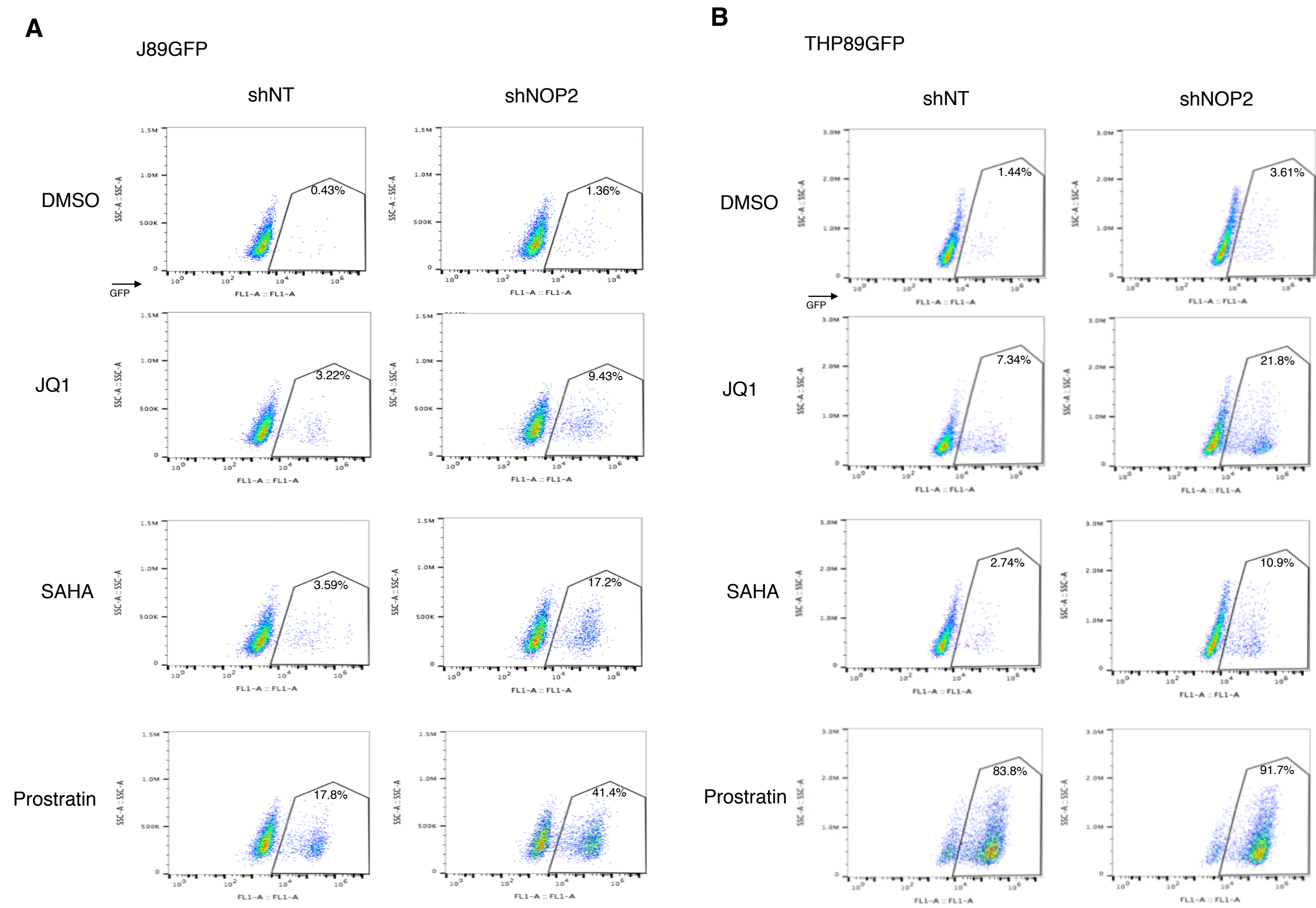

Supplement: S5 Fig — (A, B) The additional HIV-1 latency cell lines, J89GFP (A) or TH89GFP (B), were stably transduced with the indicated shRNA (shNT or shNOP2) in pAPM vector. These cells were stimulated with DMSO, JQ1 (0.5 uM), SAHA (1 uM), or Prostratin (0.5 uM), to reactivate latent HIV-1. Percentage of GFP-expressing cells was determined by flow cytometry. (PDF) [file ppat.1008430.s005.pdf]

Figure S6

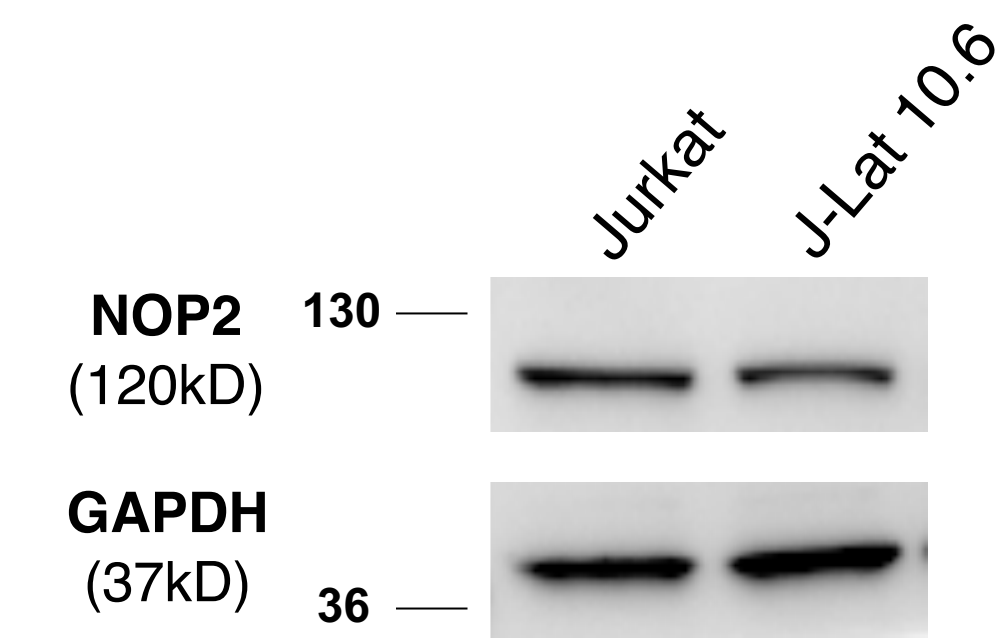

Supplement: S6 Fig — (A) Endogenous protein level of NOP2 in HIV-1 latency cell line J-Lat 10.6 and the parental Jurkat cells was measured by immunoblotting. (PDF) [file ppat.1008430.s006.pdf]
